# Structuring supramolecular hyaluronan hydrogels via peptide self-assembly for modulating the cell microenvironment

**DOI:** 10.1016/j.mtbio.2023.100598

**Published:** 2023-03-02

**Authors:** Yichen Yuan, Yejiao Shi, Jayati Banerjee, Amin Sadeghpour, Helena S. Azevedo

**Affiliations:** a School of Engineering and Materials Science & Institute of Bioengineering, Queen Mary University of London, London, E1 4NS, UK; b Zhejiang Lab, Hangzhou, 311121, Zhejiang, PR China; c Institute of Translational Medicine, Shanghai University, Shanghai, 200444, PR China; d School of Food Science and Nutrition, University of Leeds, Leeds, LS2 9JT, UK; e i3S - Instituto de Investigação e Inovação em Saúde, Universidade do Porto, Portugal; f INEB - Instituto de Engenharia Biomédica, Universidade do Porto, Rua Alfredo Allen 208, 4200-180, Porto, Portugal

**Keywords:** Amphipathic peptides, Self-assembly, Hyaluronan, Supramolecular structured hydrogels, Stem cell spheroids, ECM, extracellular matrix, HA, hyaluronan, MSC, mesenchymal stem cell

## Abstract

The use of synthetic extracellular matrices (ECMs) in fundamental *in vitro* cell culture studies has been instrumental for investigating the interplay between cells and matrix components. To provide cells with a more native environment *in vitro*, it is desirable to design matrices that are biomimetic and emulate compositional and structural features of natural ECMs. Here, the supramolecular fabrication of peptide-hyaluronan (HA) hydrogels is presented as potential ECM surrogates, combining native HA and rationally designed cationic amphipatic peptides [(KI)_n_K, lysine (K), isoleucine (I), n ​= ​2–6] whose mechanical properties and microstructure are tunable by the peptide sequence. (KI)_n_K peptides adopt β-sheet configuration and self-assemble into filamentous nanostructures triggered by pH or ionic strength. The self-assembly propensity of (KI)_n_K peptides increases with the sequence length, forming single phase hydrogels (shorter peptides) or with phase separation (longer peptides) in presence of the anionic polyelectrolyte HA through electrostatic complexations. The gel phase formed in (KI)_n_K-HA complexes exhibits viscoelastic behavior and triggers the formation of human mesenchymal stem cell (MSC) spheroids which disassemble over the time. It is anticipated that these (KI)_n_K-HA hydrogels with tunable physical and biochemical properties offer a promising platform for *in vitro* applications and in stem cell therapy.

## Highlights

• Cationic amphipathic peptides [(KI)__n__K] promote the supramolecular crosslinking of native hyaluronan.
• The mechanical and structural properties of resulting peptide-hyaluronan hydrogels are controlled by the peptide sequence.
• The structured hydrogels direct cell interactions and trigger spheroid formation of mesenchymal stem cells.

## Graphical abstract

**Figure:**
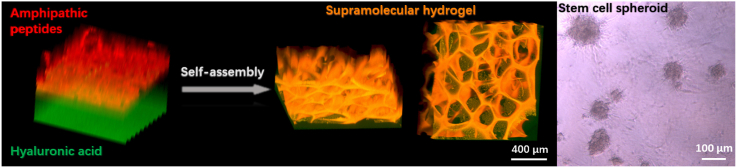

## 1 Introduction

The extracellular matrix (ECM) of tissues contains multiple proteins and glycosaminoglycans (GAGs) that not only physically support cells but also regulate their activities. Mimicking this intricate network has been a major goal in biomaterials engineering. Hyaluronic acid or hyaluronan (HA) is an essential GAG of the ECM mediating activities in cellular signaling, wound repair, morphogenesis and matrix organization, which exists either as a free GAG or non-covalently attached to proteoglycans in the ECM [1,2]. Unsurprisingly, HA has attracted large interest as building block for creating biomaterials, but the water solubility of native HA leads to its fast degradation and quick clearance *in vivo* [3]*.* As such, HA chemical modification is required to enable chain crosslinking and generate HA-based hydrogels with improved stability and functionality and tailorable mechanical properties. However, chemical functionalization of the HA backbone often requires time-consuming multi-step and less green chemical processes, and the use of non-biocompatible crosslinking agents which might raise safety concerns and limit their biomedical usage [4]. In addition, the extent and type of HA modification is known to influence its binding to cell-surface receptors, such as CD44, the major HA receptor [5]. Peptides are also popular choices to engineer synthetic ECMs as they can be designed to self-assemble into nanostructures with defined shape (e.g. nanofibers) through non-covalent interactions and display desired functionality to control cell functions (e.g. cell adhesion, proliferation, differentiation) [6]. Herein, combining HA with self-assembling peptides is expected to partially mimic the composition and architecture of the ECM to reconstruct cellular microenvironments *in vitro*.

The bottom-up approach based on the self-assembly of small molecules and polymers offers a simple, cost-effective and eco-friendly method to fabricate biomaterials without chemical modification [7]. Self-assembling peptide-polymer hydrogels have been described using fluorenylmethoxycarbonyl diphenylalanine (Fmoc-FF) with konjac glucomannan [8], alginate [9,10] and HA [11,12], but the polymer component was not actively involved in the self-assembly process, acting mainly as a filler of the peptide nanofibrous network. Yet, the self-assembly of charged polymers with small peptides of opposite charge has been shown to result in the formation of supramolecular materials with diverse forms, such as sacs [13,14], membranes [15,16], microcapsules [17] and hydrogels [[18], [19], [20]]. For example, HA was found to trigger the self-assembly of cationic peptide amphiphiles (PAs, e.g. C_16_V_3_A_3_K_3_), leading to hierarchically structured sacs and membranes [13]. Similarly, supramolecular hybrid membranes were also generated when combining HA with self-assembling multi-domain peptides (MDPs, i.e. peptides with alternated hydrophobic and hydrophilic residues terminated by charged residues) [15,16]. The formation of a diffusion barrier and phase separation normally occurs in these peptide-polyelectrolyte system through the combination effect of polyelectrolyte viscosity and strong propensity of the self-assembling peptides to form β-sheet structures. Interestingly, phase separation and hydrogel formation has also been observed in cationic surfactant-HA system, where self-assembled surfactant micelles bind strongly to HA chains via electrostatic interactions, leading to reduced overall charge and decreased hydrophilicity of the resulting complex. Surfactants with lower critical micelle concentration (cmc) showed stronger binding to HA which was essential for the hydrogel formation [21,22]. It is postulated that the self-assembly ability of cationic amphipathic peptides, via controlled β-sheet propensity, may also influence their interaction with HA. To explore this hypothesis, a supramolecular system composed of native HA and five rationally designed peptides ((KI)_n_K) with different self-assembly propensity is investigated in this work to fabricate biomimetic nanofibrous peptide-HA hydrogels with tuneable structural and mechanical properties by single step self-assembly. The presence of unmodified HA in the hydrogels is a major advantage and of biological relevance, providing a more realistic environment to probe its role in fundamental *in vitro* studies. The (KI)_n_K peptides are positively charged and composed of alternating hydrophilic and hydrophobic residues which are known to self-assemble into β-sheet fibrils [23]. Varying the length is expected to control their self-assembly propensity and interaction with HA, thus generating nanofibrous (KI)_n_K-HA hydrogels with distinct structural organization. Compared to commercially available Matrigel®, used as standard for 3D cell culture and obtained from mouse tumours, the (KI)_n_K-HA supramolecular hydrogels will also provide a biomimetic environment, composed of an ECM polymer and nanofibrous peptides, while being more reproducible (synthetically produced with minimal batch-batch variability) to facilitate and expand their applicability for 3D stem cell and organoid cultures.

HA-based hydrogels are known to favor cell spheroid formation due to its non-adhesive nature [24], while supramolecular amphipathic peptide hydrogels were also shown to support the formation of spheroids of stem [25] and cancer [26] cells. Mesenchymal stem cells (MSCs) have been regarded as a promising cell source for cell-based tissue repair and regeneration [27]. MSCs differentiate into not only mesenchymal lineages, which give rise to bone, cartilage, fat, and muscle, but also non-mesenchymal lineages, including neurons and astrocytes [28]. MSCs are conventionally maintained by 2D adherent cell culture *in vitro*, but 3D multicellular MSC spheroids often exhibit enhanced anti-inflammatory effects, improved differentiation capacity and increased cell survival after transplantation, with the advantage of promoting cell–cell communication, as well as increased secretion of pro-angiogenic factors and ECM components when compared to the traditional 2D monolayer cell culture [29,30]. Diverse technologies have been reported to generate MSC spheroids, where hanging drop and pellet culture are among the most popular methodologies, but some dynamic techniques were also utilized which require external forces (e.g. centripetal force) to induce cell aggregation [31]. Additionally, non-adhesive biomaterials are promising for promoting MSC spheroid formation. For example, surfaces coated with polycationic chitosan can efficiently generate MSC spheroids with improved stemness [32,33]. In particular, hydrogel-based MSC spheroid culture has received increased attention in tissue regeneration, but it often requires transferring the pre-formed MSC spheroids into the desired hydrogel [31,34], which is time-consuming and may also mechanically disrupt the MSC spheroids, while the hydrogel-supported MSC spheroid formation has been less reported. More recently, Yan et al. observed the formation of MSC spheroids on a covalently crosslinked gelatin methacrylate (GelMa)/polyethylene glycol diacrylate (PEGDA) hydrogel [35], which contain components from animal origin and are not fully biomimetic. In this work, a novel and biologically relevant method of *in situ* generating MSC spheroids on peptide-HA hydrogel is also presented, where MSCs spontaneously form spheroids directly on the hydrogel.

## 2 Materials and methods

### 2.1 Peptide synthesis and purification

Peptides were synthesized in an automated peptide synthesizer (Liberty Blue, CEM, UK) using standard solid-phase fluorenylmethyloxycarbonyl (Fmoc)-based chemistry. Synthesis was done in 1 ​mmol scale using a 4-methylbenzhydrylamine (MBHA) rink amide resin with substitution degree of 0.52 ​mmol/g (Novabiochem, Merck, UK). The coupling of the protected amino acids was done with four equivalents of Fmoc-Ile-OH, Fmoc-Lys(Boc)-OH (Novabiochem, Merck, UK), 1-hydroxybenzotriazole hydrate (HOBt, Carbosynth, UK) and N,*N*′-diisopropylcarbodiimide (DIC, Alfa Aesar, UK). Fmoc deprotections were performed with 20% piperidine (Alfa Aesar, UK) in dimethylformamide (DMF, Alfa Aesar, UK). Acetylation of the N-terminus was performed using 10% acetic anhydride (Sigma-Aldrich, UK) in DMF for 20 ​min. For the synthesis of rhodamine labelled peptides (Rhod-(KI)_4_K, Rhod-(KI)_5_K and Rhod-(KI)_6_K), rhodamine dye was attached at the peptide N-terminus, where 200 ​mg peptide-resin was swollen in 1200 ​μL DMF and 50 ​μL ​N,*N*-diisopropylethylamine (DIEA, Alfa Aesar, UK) with the addition of 10 ​mg of 5-(and 6)-carboxytetra-methylrhodamine succinimidyl ester (NHS-rhodamine, ThermoFisher, UK) followed by shaking at room temperature overnight. Peptides were cleaved from the resin using a cleavage cocktail composed of 95% trifluoroacetic acid (TFA, Alfa Aesar, UK), 2.5% Triisopropylsilane (TIS, Alfa Aesar, UK) and 2.5% ultrapure water. The peptide-bound resin was incubated with the cleavage cocktail in a reaction vessel for 3 ​h, at RT on a shaker (Stuart Wrist-Action Flask Shaker, UK). The peptide mixture was collected and excess TFA was removed by rotary evaporation. The resulting solution was transferred into cold diethyl ether (Alfa Aesar, UK), where a white peptide precipitated was formed. Peptides were collected by centrifugation, washed with cold diethyl ether, and allowed to dry overnight. Finally, peptides were dissolved in ultrapure water followed by lyophilization. For fluorescent labelled peptide samples, the peptide powder was redissolved in ultrapure water and then transferred into dialysis tubing (500 ​Da MWCO, ThermoFisher, UK) in which dialyzed against 100 ​mM NaCl (Sigma-Aldrich, UK), followed by dialysis against distilled water and lyophilization.

Peptides were purified by preparative reverse-phase high-performance liquid chromatography (RP-HPLC) using an autopurification system (2767 sample manager, 2545 binary gradient module, Waters, UK), and a reverse-phase C18 column (X-bridge Prep OBD, 5 ​μm, 30 ​× ​150 ​mm, Waters, UK). Peptides were dissolved in water/0.1% TFA solution (12 ​mg/mL), filtered before injection and eluted in a gradient of water/acetonitrile (ACN, Alfa Aesar, UK) containing 0.1% TFA with the flow rate of 20 ​mL/min. UV detector (2489 UV/Vis detector, Waters, UK) in the in HPLC system was set at 220 ​nm to detect the peptide bond, mass spectrometer detector (SQ-2 mass detector, UK) was set up for mass analysis, and MassLynx software with the FractionLynx application manager was used for peptide fraction collection. After collecting the purified fractions, ACN was removed by rotary evaporation before lyophilization to obtain peptides in the powder form. The purity and hydrophobicity of peptides were analyzed by analytical HPLC (Alliance HPLC System, Waters, UK) with a reverse-phase C_18_ column (X-bridge, 3.5 ​μm, 4.6 ​× ​150 ​mm, Waters, UK) using water/0.1% TFA solution with the flow rate of 1 ​mL/min. 100 ​μL of peptide solution (1 ​mg/mL) was injected and analyzed as described for the preparative HPLC.

### 2.2 Determination of peptide pK_a_ by titration of peptide solutions

100 mM NaOH (Sigma-Aldrich, UK) solution was added to a 1 ​mL peptide aqueous solution with a 1 ​mM starting concentration in 1 ​μL steps. After each NaOH addition, vortex was used to agitate the sample and enable homogenous mixing. The pH was then measured using a pH meter (FiveGo Cond meter F3, Mettler-Toledo Ltd, UK).

### 2.3 Determination of surface charge by zeta potential measurements

The degree of the stability of charged peptide aggregates was measured by Zetasizer (Nano ZS series, Malvern Panalytical, UK). Peptides were dissolved at 1 ​mM in ultrapure water and the pHs were adjusted by ammonium hydroxide (NH_4_OH). Zeta potential measurements were carried out immediately after the pH adjustment. Three repeated measurements were conducted for each sample.

### 2.4 Analysis of peptide conformation by circular dichroism (CD)

Peptide solutions for CD measurements were prepared at 0.1 ​mM concentration in ultrapure water and the pHs were adjusted to 7, 9, 9.5, 10 and 11 by adding NH_4_OH (Alfa Aesar, UK), confirmed in the pH meter. Peptide solutions were incubated overnight before the measurement was performed in a Chirascan CD spectrometer (Applied Photophysics, UK) under a continuous flow of nitrogen at a constant pressure. Far-UV spectra were recorded at 25 ​°C from 190 to 270 ​nm in a quartz cuvette with 1 ​mm path-length. Each presented spectrum is an average of 3 spectra.

### 2.5 Analysis of peptide conformation by fourier-transform infrared (FTIR) spectroscopy

Stock 20 ​mM peptide solutions were prepared by dissolving peptides in D_2_O (Sigma-Aldrich, UK). To study the peptide self-assembly at different ionic strength, stock peptide solutions (pH 7) were diluted to 10 ​mM using equal volume of NaCl solutions (in D_2_O). The final NaCl concentrations in the peptide solutions were 0, 50, 100, 200, 300 and 400 ​mM. Solutions were analyzed using a Brukler Tensor 27 FTIR spectrometer equipped with a diamond attenuated total reflection (ATR) accessory. Absorbance spectra were obtained with a 4 ​cm^−1^ resolution and D_2_O was used as the background.

### 2.6 Analysis of peptide assemblies by transmission electron microscopy (TEM)

Peptides were dissolved in deionized water to obtain the concentration of 1 ​mM and the pH was adjusted to 11 using NH_4_OH. Samples for TEM analysis was prepared by placing a drop of peptide solution directly on the 400-mesh carbon-coated copper TEM grid (Agar Scientific, UK). For negative staining, a drop of a 2 ​wt% uranyl acetate (Agar Scientific, UK) aqueous solution was placed on the samples. The excess solution was wiped away with a piece of filter paper, and the sample was allowed to dry under ambient conditions for 3 ​h. All images were collected with a JEOL 1230 transmission electron microscope at 100 ​kV (JEOL, USA) with a SIS Megaview III wide angle CCD camera. The images were analyzed in ImageJ software to determine the nanofiber diameter.

### 2.7 Supramolecular peptide-HA complex formation

To explore the interaction between (KI)_n_K peptides with HA, 1 ​wt% or 2 ​wt% of (KI)_n_K aqueous solutions (25 ​μL, pH 7) were added on top of 2 ​wt% HA aqueous solutions (25 ​μL, pH 7) unless otherwise stated. Sodium hyaluronate with molecular weight of 2 ​MDa (Lifecore Biomedical Inc, USA) was used for these experiments. All peptide-HA complexes were incubated at room temperature overnight before further characterization.

### 2.8 Kinetics of supramolecular formation of peptide-HA complexes by confocal laser scanning microscopy (CLSM)

To monitor the formation of (KI)_n_K-HA complex under CLSM, fluorescent labelled HA (fluorescein-HA) and peptide (Rhod-(KI)_n_K) were used. The synthesis of Rhod-(KI)_n_K was described in peptide synthesis. Fluorescein-HA was obtained using EDC chemistry [36]. 50 ​mg HA was dissolved in 20 ​mL ultrapure water and then mixed with a solution of 5 ​mg of fluoresceinamine (Sigma-Aldrich, UK) in 20 ​mL of DMF. Next, 100 ​mg of NHS (Sigma-Aldrich, UK) was added, and the solution pH was adjusted to 4.75 with 0.01 ​M HCl. Finally, 50 ​mg of EDC (Sigma-Aldrich, UK) was added maintaining the solution pH at 4.75. After 12 ​h, the solution was transferred to dialysis tubing (2000 ​Da MWCO, Sigma-Aldrich, UK) and dialyzed exhaustively against 100 ​mM NaCl for 2 days, followed by dialysis against distilled water for another 2 days and then lyophilization.

Individual aqueous solutions (2 ​wt%) of the hydrogel components were first prepared by mixing 10% fluorescein-HA with 90% of native HA and 1% Rhod-(KI)_n_K was combined with non-labelled peptide. 25 ​μL of HA solution was first added into a plastic ring adhered to a glass slide and then 25 ​μL of peptide solution was added on the top. The sample was immediately observed under CLSM (Zeiss LSM710, Germany) and images were taken at 15 and 60 ​min, and after overnight incubation. 3D structure was reconstructed using Zeiss Data analysis Workstation for LSM 710.

### 2.9 Nanostructural analysis of peptide-HA complexes by small angle x-ray scattering (SAXS)

SAXS experiments were performed on beamline I22 at the diamond light source (DLS) synchrotron (Didcot, UK) [37]. The energy of the beam was 12.4 ​keV, corresponding to an X-ray wavelength of 0.1 ​nm. Makrolon capillaries (Precision Extrusion Inc, USA) were used as the sample holders. The samples were prepared by first injecting 10 ​μL HA solution (2 ​wt%, pH 7) and then 10 ​μL peptide solution (1 ​wt% or 2 ​wt%, pH 7) in capillaries and incubated overnight, where the interface of peptide and HA solution was analysed. The sample-to-detector distance was fixed to 6.23 ​m. Data are presented as a function of *q = 4π* sin *θ/λ*, where *θ* is the scattering angle and *λ* is the wavelength of the incident photons. The scattering of capillary with and without solvent (water) were also collected and subtracted from the corresponding data.

### 2.10 Morphological examination of peptide-HA complexes by scanning electron microscopy (SEM)

The micro/nanostructure of the obtained peptide-HA complexes and hydrogels was analyzed by SEM. Peptide-HA hydrogel samples were fixed in 2.5% glutaraldehyde for 1 ​h at 5 ​°C. Subsequently, sequential dehydration in increasing ethanol concentrations (20%, 40%, 60%, 75%, 90%–100%, sample incubation at each concentration is 15 ​min) was conducted. To avoid collapse of the gel structure, critical point dryer (EMS 850, Electron Microscopy Sciences, USA) was used to remove ethanol. Prior to observation, samples were coated with a gold layer for 1 ​min and imaged on a field emission scanning electron microscope (FEI Inspect F, The Netherlands) using 5.0 ​kV beam.

### 2.11 Rheological analysis of solutions, complexes and hydrogels

The rheological properties of peptide-HA complexes were first assessed by performing an oscillatory amplitude sweep, from 0.01% to 100%, at a fixed frequency of 1 ​Hz using a Discovery Hybrid Rheometer (TA instruments, USA) with an 8 ​mm parallel plate at room temperature. Following the amplitude sweep, samples were subjected to a frequency sweep at a strain from the linear viscoelastic region (LVR) of the amplitude sweep. The dehydration of hydrogel was prevented by addition of water at the exposed sides of the gel.

### 2.12 Cell maintenance and materials sterilization

Human mesenchymal stem cells (MSCs, passage number 4 and 5, PromoCell GmbH, Germany) were used for the cell culture studies. Cells were cultured in 75 ​cm^2^ flasks at density of 5 ​× ​10^3^ ​cells cm ^−2^ and were maintained in DMEM culture medium (Dulbecco's Modified Eagle Medium, low glucose, GlutaMAX supplement, Gibco, Thermo Fisher, UK) supplemented with 10% fetal bovine serum (FBS), 1% Penicillin-Streptomycin. Flasks were incubated at 37 ​°C, 5% CO_2_. Medium was refreshed every three days, and cells were trypsinized when reached 80% confluency. Sterilization of hydrogel components for cell culture was done by dissolving HA or alginate (alginic acid sodium salt, medium viscosity, sourced from brown algae, Sigma-Aldrich, UK) in ultrapure water, filtering the polymer solution through a 0.22 ​μm sterile filter, followed by lyophilization in sterile falcon tubes (TubeSpin® Bioreactors, Switzerland). Peptide powders were sterilized by UV exposure for 15 ​min.

### 2.13 Cell culture on the hydrogels

Hydrogels were prepared in 96-well plates under sterile environment (cell culture hood) by adding 25 ​μL 1 ​wt% or 2 ​wt% peptide ((KI)_5_K or (KI)_6_K, pH 7) aqueous solution on the top of 25 ​μL 2 ​wt% HA solution (pH 7) and incubated at room temperature overnight, followed by adding 200 ​μL culture media allowing for hydrogel gelation or swelling, respectively. After 1 ​h, the extra liquid around the gel was removed before adding 200 ​μL of single cell suspension on top of the gel. 10^4^ ​cells were seeded on each hydrogel. MSCs were also seeded on peptide-alginate and Ca^2+^-alginate hydrogels under the same conditions. Alginate hydrogels were used as controls where peptide-alginate hydrogel was formed by adding 25 ​μL 1 ​wt% (KI)_4_K, (KI)_5_K or (KI)_6_K solution (pH 7) on the top of 25 ​μL 2 ​wt% alginate solution (pH 7). Ca^2+^-alginate hydrogel was made by adding 25 ​μL 100 ​mM CaCl_2_ on the top of 25 ​μL 2 ​wt% alginate solution.

### 2.14 Cell viability and morphology

The viability of MSCs was assessed using LIVE/DEAD Viability/Cytotoxicity Kit for mammalian cells (Invitrogen, UK). Briefly, hydrogel samples were treated with 4 ​μM calcein AM and 2 ​μM ethidiumhomodimer-1 and incubated for 20 ​min at room temperature. Stained cells on the hydrogels were imaged using confocal microscopy on day 1, 3 and 7. The morphology of cells cultured on the hydrogels was observed using SEM and prepared as describe before. F-actin staining was also conducted to observe cytoskeleton fibres of MSCs. MSCs on the hydrogel were washed in PBS, fixed in 4% paraformaldehyde at room temperature for 20 ​min and permeabilised using 0.1 ​wt% Trition X-100 for 20 ​min. They were then stained with 1:100 Alexa Fluor phalloidin (Alexa Fluor™ 488 Phalloidin, Invitrogen, UK) and 1:1000 Dapi (Invitrogen, UK) in PBS at room temperature for 40 ​min. Samples were then washed with PBS twice before examination by CLSM and 3D reconstruction was performed using Zeiss Data analysis Workstation for LSM 710. The morphology of cells on the hydrogels was also observed by SEM and using the same procedure as described for cell-free hydrogel.

### 2.15 Statistical analysis

Data are presented as mean ​± ​standard error. Statistical differences were obtained with MATLAB R2019b software using two-tailed Student's t-tests (comparison between two experimental groups), or using one-way ANOVA with Post Hoc Tukey HSD test (comparisons among more groups). ∗∗∗∗ ​= ​p ​< ​0.0001, ∗∗∗ ​= ​p ​< ​0.0002, ∗∗ ​= ​p ​< ​0.0021, and ∗ ​= ​p ​< ​0.0332.

## 3 Results and discussion

### 3.1 Peptide design and characterization

**Fig. 1:**
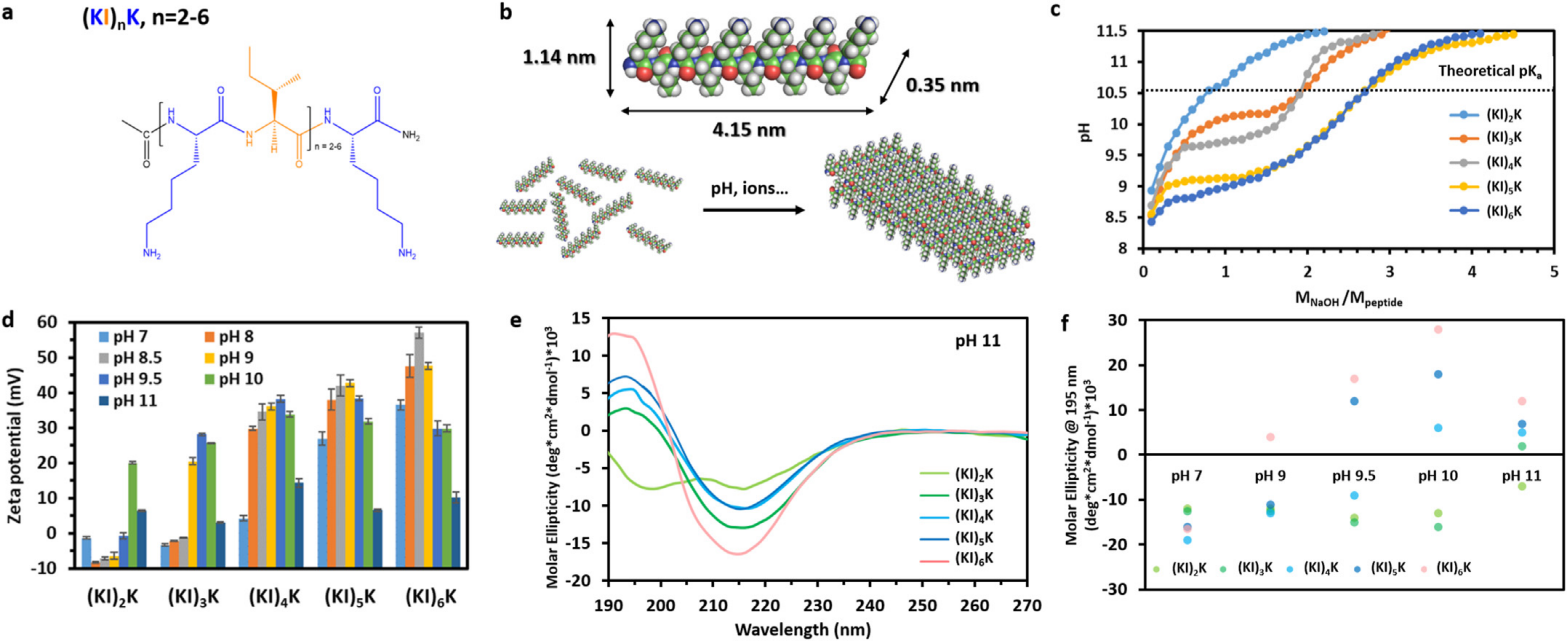
(a) Generic chemical structure of (KI)_n_K peptides. (b) Schematic representation of peptide self-assembly into bilayer (dimer) β-sheet structure shown for (KI)_5_K. (c) Titration curves of (KI)_n_K peptide solutions (1 ​mM) for determining their *pK*_*a*_. (d) Zeta potential of (KI)_n_K peptide solutions (1 ​mM) at varied pHs. Standard deviation is indicated by error bars and measurements were performed in triplicate. (e) CD spectra of (KI)_n_K peptide solutions (0.1 ​mM) at pH 11. (f) Molar ellipticity at 195 ​nm in CD spectra of (KI)_n_K peptides at different pHs.

(KI)_n_K peptides possess variable number of a repeating unit containing alternating hydrophilic cationic (lysine, K) and hydrophobic (isoleucine, I) residues, as depicted in Fig. 1a. (KI)_n_K peptides could self-assemble into amyloid-like nanofibers, when charges are screened under increased pH and ionic strength, with hydrophobic residues buried in the interior while the hydrophilic residues are exposed to the aqueous medium (Fig. 1b). Increasing the number of the repeating hydrophobic-hydrophilic unit (*n*) is known to generate peptides with stronger driving forces for β-sheet formation due to enhanced hydrogen bonding [23], but as the number of like-charge increases with the peptide length, the repulsive forces may also prevent the self-assembly. Therefore, studying how these two counteracting forces affect the self-assembly of (KI)_n_K peptides can reveal new insights to design self-assembling peptides with tunable properties. In this study, five peptides (KI)_2_K, (KI)_3_K, (KI)_4_K, (KI)_5_K and (KI)_6_K were synthesized and their self-assembly behavior was investigated under different environmental conditions. The pH-dependence of peptide self-assembly can be estimated based on the theoretical *pK*_*a*_ of containing amino acids, but different chemical microenvironments can lead to a shift of *pK*_*a*_ [38]. The ionic groups in (KI)_n_K peptides are on the side chain of lysine residues, whose theoretical *pK*_*a*_ is 10.53. Therefore, it is assumed that the peptide self-assembly is not favored at pH below 10.53 due to electrostatic repulsion. Interestingly, the titration curves (Fig. 1c) indicate that (KI)_n_K peptides show increased *pK*_*a*_ shifts. The titration curve of (KI)_2_K showed a reduced pH change at around pH 10.5, indicating a transition at this pH, in good agreement with the theoretical *pK*_*a*_ value, while in (KI)_3_K and (KI)_4_K a ‘plateau’, with less pronounced pH change, was observed below their theoretical *pK*_*a*_, where the *pK*_*a*_-like transition for (KI)_3_K was at around pH 10.1 and at pH 9.7 for (KI)_4_K. (KI)_5_K and (KI)_6_K exhibited greater *pK*_*a*_ shifts towards lower pH as *pK*_*a*_-like transitions were found to be at around pH 9.1 and 8.8, respectively. In (KI)_n_K peptides, the proximity of hydrophobic (I) and hydrophilic charged (K) residues promotes hydration competition between them, which results in the elevated nonpolar-polar repulsive free energy of hydration inducing the *pK*_*a*_ shift [39]. In addition, the ratio of hydrophobic residues to the total amino acid number in (KI)_2_K, (KI)_3_K, (KI)_4_K, (KI)_5_K and (KI)_6_K peptides is 0.40, 0.43, 0.44, 0.45 and 0.46, respectively. Thus, the overall peptide hydrophobicity is expected to increase as the number of the repeating unit (n) becomes higher, which is also confirmed by the RP-HPLC profiles (Table S1, Fig. S1, Supplementary data) where longer (KI)_n_K peptides show longer retention times (eluted at higher percentage of non-polar solvent). Since the greater peptide *pK*_*a*_ shift is expected to be found in a more hydrophobic background [38,39], it is suggested that the lower *pK*_*a*_ of longer (KI)_n_K peptides might be ascribed to its increased hydrophobicity. The effect of pH on peptide self-assembly was further investigated by measuring the zeta potential of peptide aqueous solutions at different pHs (Fig. 1d). (KI)_2_K, (KI)_3_K and (KI)_4_K peptides showed a very low absolute zeta potential at neutral pH, which suggests the peptide molecules were not aggregating and remained as monomers in these conditions. The higher zeta potential values of (KI)_5_K (26.9 ​± ​1.9 ​mV) and (KI)_6_K (36.5 ​± ​1.5 ​mV) indicates relatively stable peptide colloids with high number of positive charges at neutral pH. Zeta potential of each peptide declines at pH close to their individual *pK*_*a*_, showing the deprotonation of –NH_3_^+^ on the lysine side chains, and continuously decreases with rise in pH.

The secondary structures of (KI)_n_K peptides with pH change were then evaluated by circular dichroism (CD) spectroscopy. At pH 7, the peptides show a minimum at 195 ​nm corresponding to random coil structure (Fig. S2, Supplementary data). At pH 11 (when all peptides pass over their *pK*_*a*_), the CD spectra of all peptides, except (KI)_2_K, displayed a characteristic profile of β-sheet with positive band at around 194 ​nm and negative band at 216 ​nm, while (KI)_2_K showed the weak negative bands at both 199 and 216 ​nm indicating a mixture of random coil and β-sheet structures (Fig. 1e) [40]. The molar ellipticity at 195 ​nm of (KI)_n_K peptides at varied pHs is shown in Fig. 1f revealing the peptide secondary structure changes as pH increases from 7 to 11. At 195 ​nm, there is a characteristic negative minimum of random coil while β-sheet structure exhibits positive maximal molar ellipticity. The CD spectra observed for (KI)_n_K peptides show that in general the peptides behaved as random coils at pH below their individual *pK*_*a*_ and gradually formed a β-sheet structure as pH increased, where the conformation transition could be observed at around their *pK*_*a*_. (KI)_n_K peptides are capable of forming β-sheet structures at basic pH due to the reduced intermolecular electrostatic repulsion, encouraging the self-assembly, and longer (KI)_n_K peptides form β-sheet structures at relative lower pHs corresponding to their *pK*_*a*_ value. Increasing ionic strength would also induce peptide self-assembly. NaCl was used to adjust the ionic strength as Cl^−^ could function as monovalent counterion to screen the positive charges on the side chain of lysine residues. In Fourier-transform infrared (FTIR) spectra (Fig. S3, Supplementary data), the formation of β-sheet structures was observed with increasing NaCl concentration at pH 7. (KI)_2_K peptide showed no evident β-sheet formation with the increasing NaCl concentration up to 400 ​mM, and the minimal NaCl concentration required for the β-sheet assembly for (KI)_3_K, (KI)_4_K, (KI)_5_K and (KI)_6_K was 400, 300, 100 and 50 ​mM, respectively. Since the β-sheet propensity of ionic self-complementary peptides is known to increase with the peptide hydrophobicity [41,42], it is assumed that increased ratio of hydrophobic-hydrophilic residues can trigger β-sheet assembly of (KI)_n_K peptides at lower ionic strength in a synergistic effect. In general, the self-assembly propensity of (KI)_n_K peptides increases with the sequence length though longer peptides carries more like-charges, which results in more intense repulsive electrostatic forces.

**Fig. 2:**
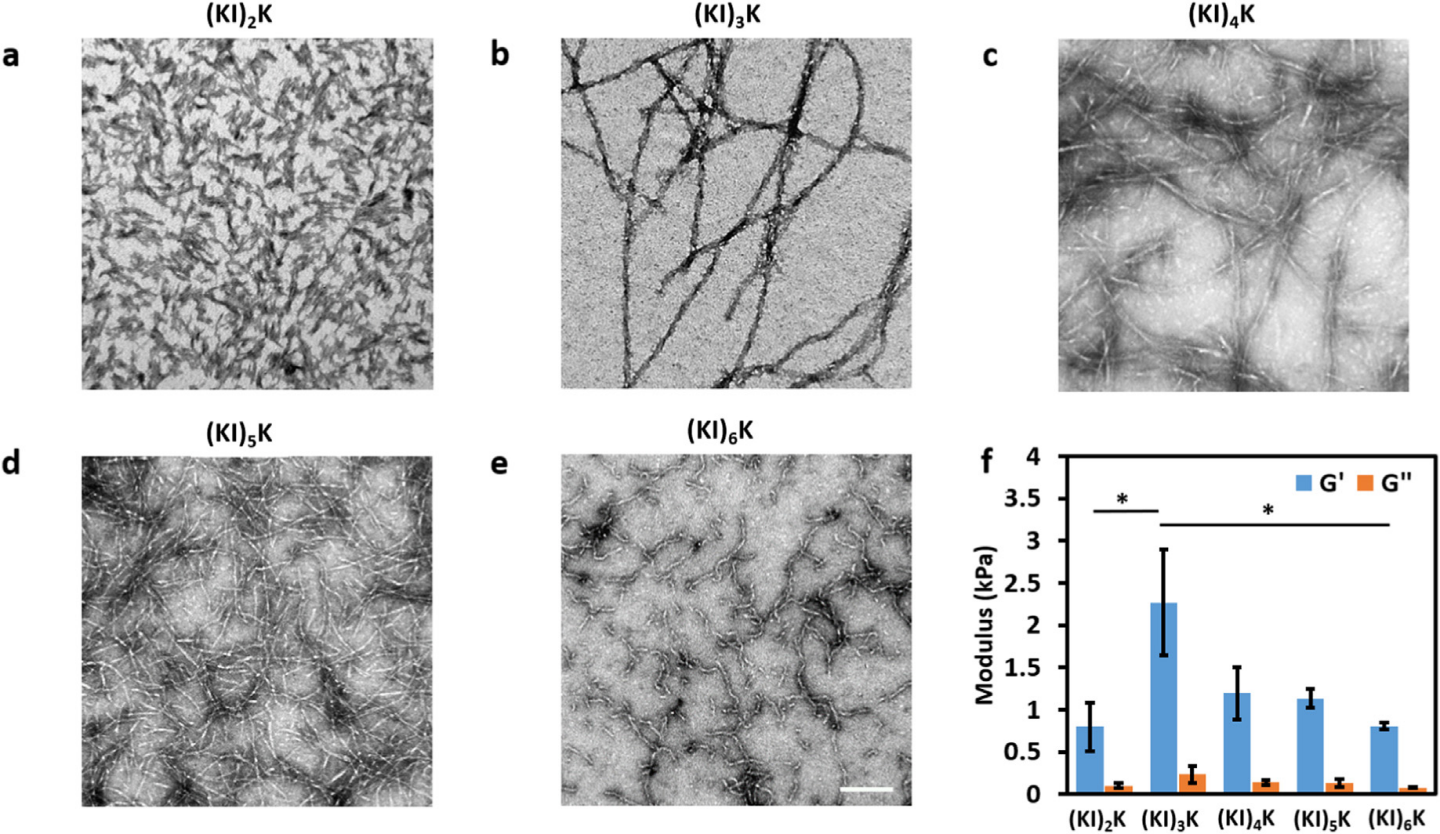
TEM micrographs of (a) (KI)_2_K, (b) (KI)_3_K, (c) (KI)_4_K, (d) (KI)_5_K and (e) (KI)_6_K peptides at pH 11 (1 ​mM, Scale bar ​= ​100 ​nm). (f) Storage modulus (G′) and loss modulus (G″) of (KI)_n_K peptide hydrogels (1 ​mM) at pH 11 obtained from frequency sweep at 1 ​Hz. Standard deviation is indicated by error bars and measurements were performed in triplicate (∗ ​= ​*p* ​< ​0.0332).

Transmission electron microscopy (TEM) was used to confirm the morphology of the supramolecular peptide assemblies at basic conditions (pH 11, Fig. 2a–e). pH 11 was chosen for these studies because it is above the *pK*_*a*_ of all peptides and to ensure charges are screened to favor their self-assembly. (KI)_2_K peptide forms very thin fibrils which are difficult to distinguish from each other (being challenging for quantification) due to the limited β-sheet forming capability. The quantification of the diameter of (KI)_3_K, (KI)_4_K, (KI)_5_K and (KI)_6_K fibrils was performed. (KI)_3_K and (KI)_4_K formed long and twisted fibers with an average width of 12.4 ​± ​3.0 ​nm and 7.6 ​± ​3.0 ​nm, respectively, which appears to be bundles of smaller fibrils. Entangled long nanofibrils could also be found in (KI)_5_K, while (KI)_6_K self-assembled into short worm-like fibril structures. The average fibril diameter of (KI)_5_K and (KI)_6_K dramatically decreased to 4.3 ​± ​0.8 ​nm and 4.6 ​± ​1.0 ​nm, which is consistent with the estimated length of linear peptide chain (one amino acid is considered to be around 0.4 ​nm [43]). Repulsive electrostatic interactions are known to reduce the fibril bundling ability and the fibril bundling effect is less evident as the peptide length increases. Though longer peptides have lower *pK*_*a*_, (KI)_5_K and (KI)_6_K peptides require more NaOH addition to reach high pH, as shown in the titration curve. Thus, it is assumed that the deprotonation process of (KI)_5_K and (KI)_6_K peptides after *pK*_*a*_-like transition is much slower than for shorter peptides, suggesting that some lysine residues on (KI)_5_K and (KI)_6_K peptides may remain protonated even at high pH. Such slow deprotonation process is also observed in the titration of polyelectrolytes, where charge-charge repulsion along the polymer chain influences deprotonation [44]. The mechanical properties of supramolecular peptide assemblies were assessed by measuring the viscoelasticity of peptide hydrogels at pH 11 (Fig. 2f). All peptide samples displayed much larger *G′* than *G″* independent of frequency, indicating the formation of a hydrogel, where (KI)_3_K hydrogel is the most rigid one, with *G′* close to 2 ​kPa, followed by (KI)_4_K and (KI)_5_K, exhibiting *G′* around 1 ​kPa, while (KI)_2_K and (KI)_6_K gels are the weakest, whose G’ are only around 0.8 ​kPa. (KI)_3_K, (KI)_4_K and (KI)_5_K gels are relatively stronger probably because they formed longer fibrils leading to the enhanced fibril entanglement. The thicker nanofibers formed by (KI)_3_K peptide may also contribute to highest G' observed. In general, it was found that increasing the length of (KI)_n_K peptides leads to the stronger β-sheet forming propensity, but it would also reduce the bundling ability of the self-assembled nanofibrils.

### 3.2 Supramolecular peptide-HA hydrogel

**Fig. 3:**
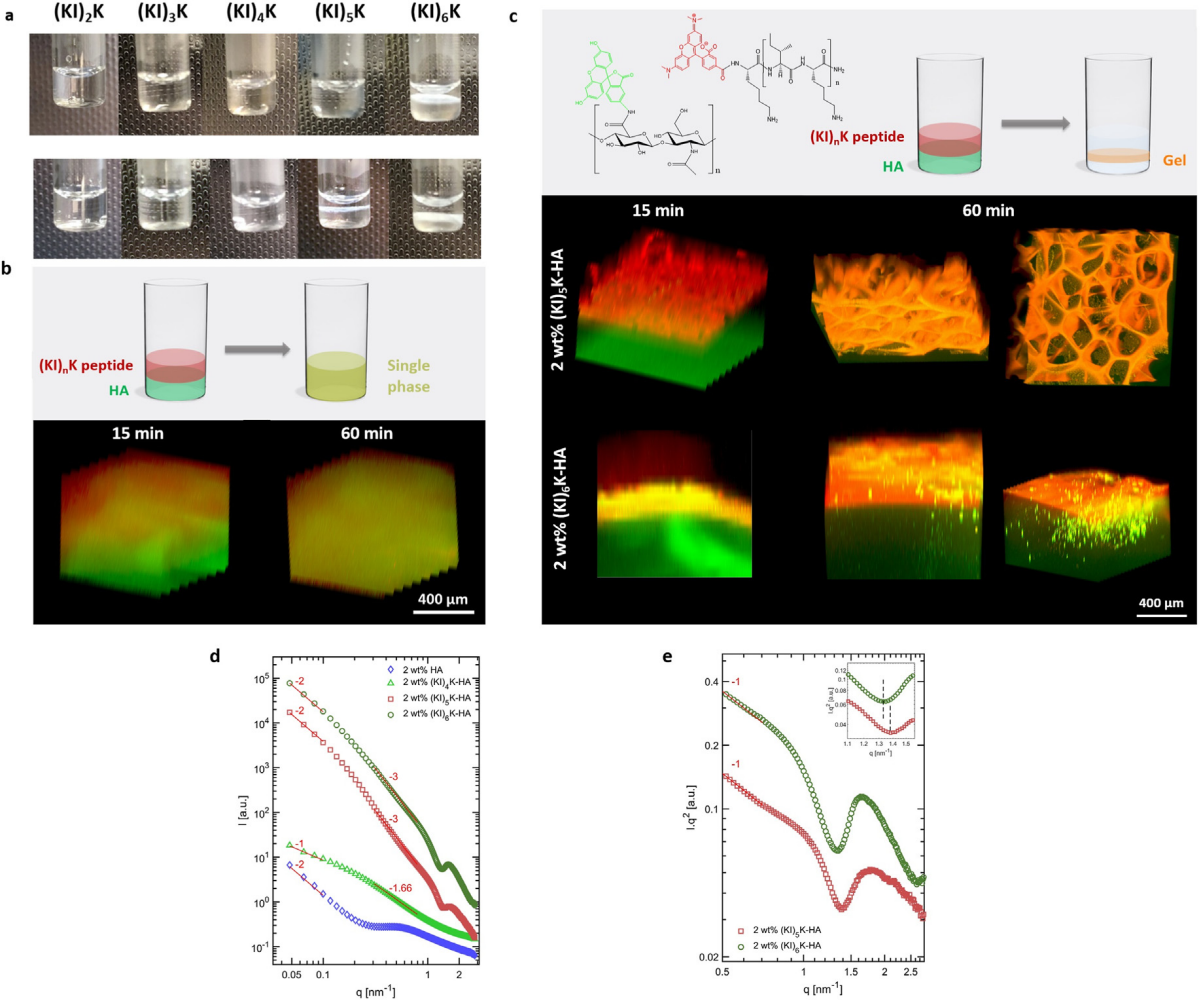
(a) Supramolecular (KI)_n_K-HA complexes formed in a glass vial when (KI)_n_K peptide solutions (1 ​wt%, top row, and 2 ​wt%, bottom row) are added on top of HA solution (2 ​wt%) and incubated overnight. (b) Schematic representation of the formation of single-phase (KI)_n_K-HA system and reconstructed CLSM 3D images used to study the assembly kinetics of single-phase 2 ​wt% (KI)_4_K-HA complex. (c) Schematic representation of (KI)_n_K-HA hydrogel formation and reconstructed CLSM 3D images used to study the assembly kinetics of 2 ​wt% (KI)_5_K-HA and 2 ​wt% (KI)_6_K-HA hydrogels. (d) Double logarithmic plots of SAXS patterns obtained for single-phase 2 ​wt% HA, 2 ​wt% (KI)_4_K-HA complex, 2 ​wt% (KI)_5_K-HA and 2 ​wt% (KI)_6_K-HA hydrogels in *I* vs *q* representation. (e) Double logarithmic Kratky plots of SAXS patterns for 2 ​wt% (KI)_5_K-HA and 2 ​wt% (KI)_6_K-HA hydrogel phases.

The self-assembly of (KI)_n_K peptides with HA was investigated by casting the peptide aqueous solution (pH 7) on top of the HA solution (pH 7), where the anionic HA could screen the positive charges of (KI)_n_K peptides. As the HA solution has high viscosity (34 ​Pa ​s at the shear rate of 10 s^−1^), due to the large molecular weight (2 ​MDa), the two solutions are not expected to mix immediately. After incubation at room temperature overnight, it was found that either a single-phase homogeneous complex was observed or a phase separation occurred in the (KI)_n_K-HA self-assembling system (Fig. 3a). A gel-like phase emerged at the interface between HA and peptide solutions with 2 ​wt% (KI)_5_K, 2 ​wt% (KI)_6_K or 1 ​wt% (KI)_6_K. Hydrogels were formed for 2 ​wt% (KI)_5_K-HA and 2 ​wt% (KI)_6_K-HA self-assembling system which could be easily taken out from the low viscosity supernatant phase. Similar phenomena were also observed in some cationic surfactant-HA system, where the gel phase was believed to contain all HA and the supernatant phase having a viscosity as pure solvent [22,45]. Though distinguished phases were also observed in the supramolecular 1 ​wt% (KI)_6_K-HA system, the extra liquid below the gel phase still has a high viscosity and the two phases were stuck together, indicating that there was HA remaining in the liquid. However, phase separation did not occur in the 2 ​wt% (KI)_2_K-HA, 2 ​wt% (KI)_3_K-HA and 2 ​wt% (KI)_4_K-HA systems. Similarly, only a clear single phase was found when mixing HA and 1 ​wt% (KI)_2_K, (KI)_3_K, (KI)_4_K or (KI)_5_K. Since the longer (KI)_n_K peptides have a stronger β-sheet forming propensity, whose repulsive positive charges could be more readily screened, it is plausible that phase separation can only occur in the supramolecular system with HA and longer peptides. In the cationic surfactant-HA system, phase separation was not observed for surfactants with shorter alkyl chains (i.e. with weaker self-assembly propensity) [21]. To investigate the kinetics of the supramolecular formation of the (KI)_n_K-HA complex, along with the peptide and HA distribution, rhodamine and fluorescamine dyes were conjugated to (KI)_n_K and HA, to produce fluorescent probes (Rhod-(KI)_n_K and fluorescein-HA), respectively. The masses of Rhod-(KI)_n_K peptides were confirmed by mass spectrometry (Fig. S4, Supplementary data). The kinetics of the typical (KI)_n_K-HA single-phase system are shown in Fig. 3b for 2 ​wt% (KI)_4_K-HA and Fig. S5 (Supplementary data) for 1 ​wt% (KI)_4_K-HA and 1 ​wt% (KI)_5_K-HA. No defined structure was observed at the interface after incubation, with the red and green signals overlapping, reflecting the homogeneous mixing of the two components and suggesting the relatively weak interaction between the peptide and HA. In the supramolecular 2 ​wt% (KI)_5_K-HA and 2 ​wt% (KI)_6_K-HA system, where phase separation occurred, distinct separated layers were observed (Fig. 3c). In 2 ​wt% (KI)_5_K-HA system, a porous layer forms shortly at the interface after the addition of peptide on the HA solution, and the layer continues to grow in thickness until the formation of a microporous 3D network was observed after 1 ​h. In the 2 ​wt% (KI)_6_K-HA system, the diffusion barrier-like structure could be observed during the formation of the hydrogel. In the supramolecular peptide-HA membranes and sacs previously reported, similar dense diffusion barrier was observed upon contact between the peptide and HA solutions [13,46], but a porous layer was rarely observed. It is suggested that the nano-scale aggregation of the peptide in its aqueous solution would facilitate the formation of diffusion barrier once in contact with HA [14]. As the longer (KI)_n_K peptide has a stronger self-assembly propensity, (KI)_6_K was more likely to form supramolecular nanostructures in its aqueous solution and thus a diffusion barrier could be observed during the formation of (KI)_6_K-HA hydrogel. Overnight incubation showed the homogeneous distribution of both peptide and HA for 2 ​wt% (KI)_5_K-HA hydrogel (Fig. S6, Supplementary data), because the porous interface allowed the free diffusion. However, the two components were not evenly distributed in 2 ​wt% (KI)_6_K-HA due to the formation of diffusion barrier, where the bottom and the edge of the hydrogel were more HA-rich.

Small angle X-ray scattering (SAXS) was performed to investigate the nanostructural organization of HA chains and the peptide-HA complexes. The scattering intensities as a function of the scattering vector magnitudes (q) are shown in Fig. 3d for different hydrogel samples and controls. The scattering data are represented in the so-called inverse space, or Fourier space, meaning that smaller q values in the x-axis represent the larger size scale. For 2 ​wt% HA solution, a Gaussian polymer chain scattering behavior is observed, indicated by the initial decay rate −2 (the q dependance of intensity decay follows the rule of I ​∝ ​*q*^*−2*^) for q values up to 0.2 ​nm^−1^. The 0.2 ​nm^−1^ refers to distances larger than 31 ​nm when applying the inverse space rule of 2π/q. This means that at distances superior than the limit, the polymer chain experiences no interactions and resembles more of the behavior of freely-jointed chain. In some literature, the decay rate is referred to as Porod exponent, or Porod slop, as it is equivalent to the slope of the intensity versus q in a double logarithmic plot. The scattering intensity above 0.2 ​nm^−1^ limit shows a broad peak with maximum around 0.58 ​nm^−1^, representing a 10.8 ​nm correlation length within the polymer chain [47]. Similar correlation peaks for other polymer systems in solution has been observed previously, normally at high concentrations, where chain packing or semi-crystalline domains could be formed [14]. 2 ​wt% (KI)_4_K-HA complex (single phase), also shows slightly different behavior below and above 0.2 ​nm^−1^ limit. Initially, a *q*^*−1*^ dependence of the scattering intensity (Porod exponent of −1) confirms the presence of elongated rod-like structures. The scattering decay rate then slightly increases to the Porod exponent value of −1.67, in the q-range of 0.3–0.8 ​nm^−1^. This decay rate is an indication of more elongated polymer chains due to some repulsive interactions within the chain [47]. The scattering profile also demonstrates a monotonous decrease with no broad peak, suggesting the absence of any correlations within the polymer chain or formation of β-sheet self-assemblies (Fig. 3d). The scattering analysis of the 2 ​wt% (KI)_5_K-HA and 2 ​wt% (KI)_6_K-HA gel phase confirms a different behavior. It starts with the initial Porod exponent of −2 (I ​∝ ​*q*^*−2*^) followed by even higher decay rate of −3 (I ​∝ ​*q*^*−3*^). The initial decay (below 0.2 ​nm^−1^) could be associated to Gaussian chain behavior, similar to one of pure HA. It is noteworthy that according to the scattering theory, the *q*^*−2*^ decay could also be assigned to 2D planar surfaces. However, attempts to simulate the scattering intensities with planar models were unsuccessful as the intensity fluctuations at higher q-range could not be simulated (Fig. S7, Supplementary data). It is speculated that at the scattering vector magnitudes below 0.2 ​nm^−1^, the intensity from Gaussian polymer chains dominates. However, a −3 Porod exponent emerges in both samples at higher q-range (smaller length scales), indicating the formation of aggregates, i.e. 3-dimensional structures or self-assemblies in both gels [48]. In order to achieve detailed structural insights into these self-assembled systems, the Kratky plot*, I×q*^*2*^ versus *q*, has been presented in Fig. 3e. This Kratky plot excludes, indeed, the scattering from free Gaussian polymer chains and represents the scattering from fibrillar rod-like self-assembled structures only. The Kratky plot shows a slope of −1 at medium q-range at around 0.5 ​nm^−1^, which confirms the presence of rod-like fibrillar structures, according to the scattering theory. Looking into slightly higher q values, more intensity fluctuations could be identified. Such intensity fluctuations can be associated to the electron density contrast from cross-section of self-assembled fibrillar structures. According to the theory of scattering from a monodisperse cylindrical system with cross-sectional radius of gyration (R_gc_), number of minima and maxima could be observed, with the first and second minima appearing at *3.83/R*_*gc*_ and *7.01/R*_*gc*_ [49]. Both self-assemblies in 2 ​wt% (KI)_5_K-HA and 2 ​wt% (KI)_6_K-HA gels show a minimum at q values around 1.4 ​nm^−1^. Applying the first minimum position rule to both gel systems, 2.77 ​± ​0.03 and 2.88 ​± ​0.03 ​nm were estimated for the cross-sectional radius of gyration for (KI)_5_K-HA and (KI)_6_K-HA, respectively. These values are slightly larger than the ones estimated for the (KI)_5_K and (KI)_6_K bilayer (dimer) and the fibril diameter observed in TEM (Fig. 2a–e). The differences observed are probably due to the presence of HA in the fibril and the fact that TEM was performed on dried peptide assemblies.

**Fig. 4:**
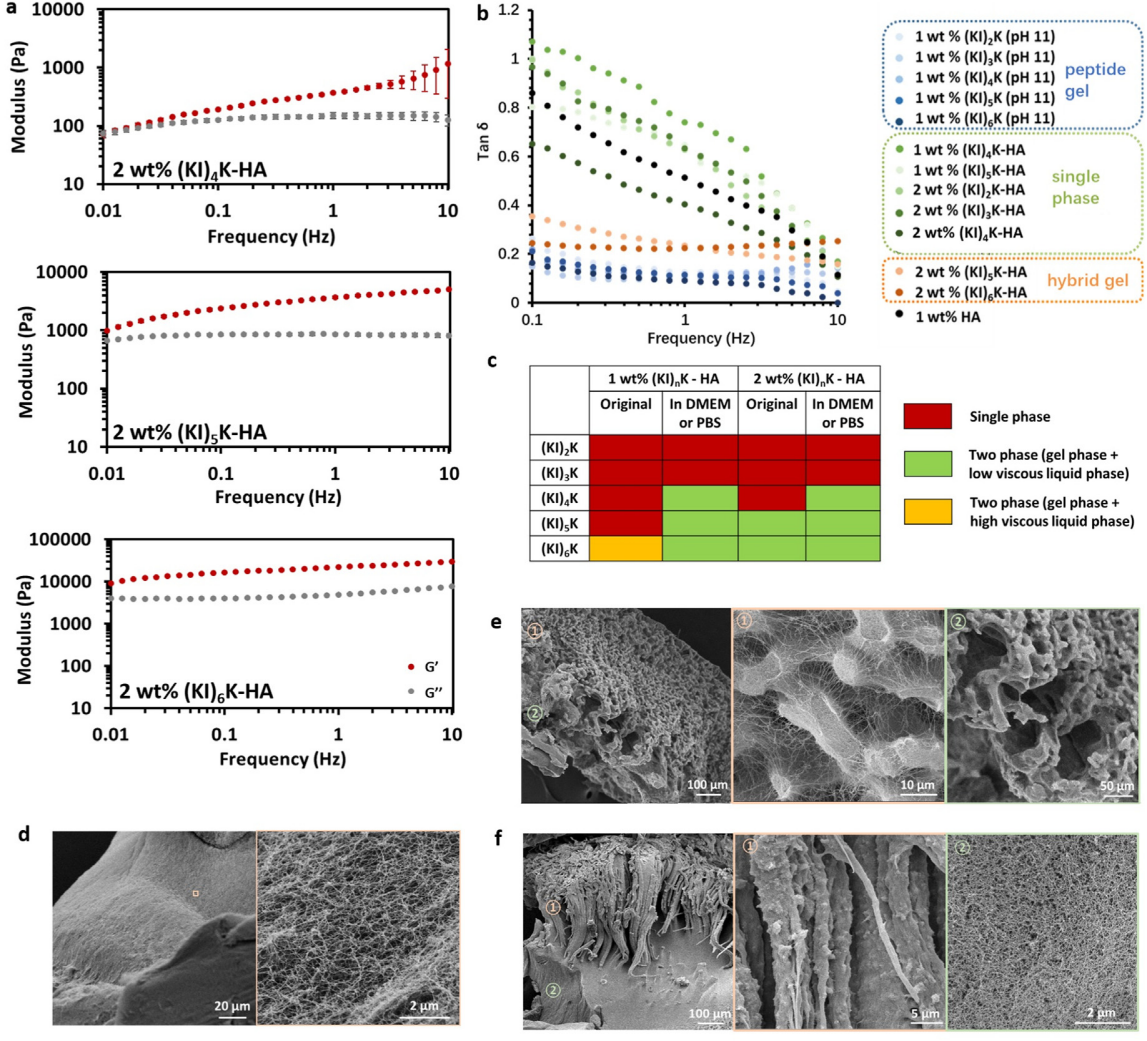
(a) Rheological analysis of 2 ​wt% (KI)_n_K-HA complexes or hydrogels. Standard deviation is indicated by error bars and measurements were performed in triplicate. (b) Tan *δ* of peptide gel, single-phase peptide-HA complex and peptide-HA hydrogel against frequency measured by rheometer. (c) The state of the supramolecular (KI)_n_K-HA complexes before and after overnight incubation in physiological-like conditions at room temperature. Cross-section SEM images displaying (d) homogeneous hydrogel of 2 ​wt% (KI)_4_K-HA complex in PBS, (e) microporous structure of 2 ​wt% (KI)_5_K-HA hydrogel, and (f) hierarchical organization of 2 ​wt% (KI)_6_K-HA hydrogel.

The mechanical properties of the (KI)_n_K-HA complexes were then investigated by conventional rheology (for the two-phase complex, only the gel-like phase was measured). 1 ​wt% (KI)_6_K-HA hydrogel was not measured since separation of the gel phase from the supernatant was not possible. All complexes with HA and 1 ​wt% (KI)_n_K peptides were found to have rheological behavior similar to pure HA, independent of the peptide sequence, indicating minor contribution of the self-assembling peptides, and with HA dominating the mechanical properties of the mixtures (Fig. S8, Supplementary data). Large molecular weight HA is known to have higher *G″* than *G′* in low frequency region, but *G′* then predominates over *G″* at higher frequencies due to the transient HA network formed by increased molecular entanglement in the short period of oscillation [50]. Single-phase complexes composed of HA and 2 ​wt% (KI)_2_K, (KI)_3_K and (KI)_4_K also exhibit HA-like rheological behavior. While the cross-over frequency (when *G’* ​= ​*G″*) for 2 ​wt% (KI)_2_K-HA and 2 ​wt% (KI)_3_K-HA was found at around 0.1 ​Hz, for 2 ​wt% (KI)_4_K-HA mixture the cross-over point was observed at lower frequency (∼0.01 ​Hz) probably due to the relatively weak peptide nanofibril entanglement turning the viscoelastic single-phase complex into a form closer to a gel (Fig. 4a, Fig. S8, Supplementary data). The rheological behavior of 2 ​wt% (KI)_5_K-HA and 2 ​wt% (KI)_6_K-HA hydrogels is significantly different from the other single-phase system, as there is a dramatic increase of both *G′* and *G″*, being also much less frequency dependent, where the contribution of self-assembling peptides dominates the mechanical properties of the hydrogels. Generally, longer (KI)_n_K peptides shifted the viscoelastic profile of the hydrogels towards lower frequencies and led to higher stiffness in 2 ​wt% (KI)_n_K-HA complex. Loss factor (Tan *δ*, *G*’’*/G’*) quantifies the balance between elasticity and viscosity, and tan *δ* of the peptide gels (peptide at pH 11), peptide-HA single-phase complex and peptide-HA gel against frequency is shown in (Fig. 4b). The profile of tan *δ* of single-phase complex is similar to HA solutions, showing the liquid-like behavior, while pristine (KI)_n_K gel has much lower tan *δ* confirming the solid-like nature. Generally, tan *δ* of supramolecular peptide-HA hydrogel is observed to be lower than HA or single-phase peptide-HA complex, but higher than the conventional supramolecular peptide hydrogel. It is assumed that the peptide-HA hydrogel mainly inherits the high viscosity from HA and elasticity from self-assembling peptide network.

The stability of (KI)_n_K-HA complexes in physiological condition (PBS or DMEM) was investigated and a summary is provided in (Fig. 4c). 1 ​wt% (KI)_6_K-HA, 2 ​wt% (KI)_5_K-HA and 2 ​wt% (KI)_6_K-HA hydrogels remained stable in these conditions, while some of liquid-like single-phase complexes (1 ​wt% (KI)_4_K-HA, 1 ​wt% (KI)_5_K-HA, and 2 ​wt% (KI)_4_K-HA) were able to transform into hydrogels with the addition of DMEM or PBS, as phase separation occurred, and all hydrogels could be easily taken out of the supernatant (Fig. S9, Supplementary data). However, none of the (KI)_2_K-HA and (KI)_3_K-HA complexes formed any kind of self-supporting hydrogel and finally dissolved in DMEM or PBS, probably due to the limited β-sheet forming ability of the peptides. It is postulated that counterions (e.g. Cl^−^ and HPO_4_^2−^) in DMEM and PBS could further screen repulsive positive charges of the peptides and finally trigger the hydrogel formation. SEM was used to examine the microstructure of the hydrogels in PBS, revealing varied structures depending on the peptide sequence. Hydrogels formed by single-phase (KI)_n_K-HA complexes have a homogeneous structure with the addition of PBS, enabled by the free diffusion of the peptide and HA, and the magnified image shows that they were composed of nanofibrous structures (Fig. 4d, Fig. S10, Supplementary data). In 2 ​wt% (KI)_5_K-HA hydrogel, large and small pores formed by thin fibrils were visible at each side of the hydrogel cross-section (Fig. 4e), while in 2 ​wt% (KI)_6_K-HA, there were two distinct regions, with the central part of the hydrogel consisted of randomly entangled nanofibrils and the oriented fiber at the edge (Fig. 4f). Furthermore, three different zones were also found in 1 ​wt% (KI)_6_K-HA hydrogel, which are amorphous layer, parallel and perpendicular aligned fiber regions (Fig. S11, Supporting Information), similar to the hierarchical organization seen in other supramolecular peptide-HA membranes [13,15,46,51]. The formation of the oriented fiber organization in the supramolecular peptide-HA hydrogel is likely to result from the formation of an early diffusion barrier, as previously suggested [13,14].

### 3.3 Formation of MSC spheroids on supramolecular peptide-HA hydrogels

**Fig. 5:**
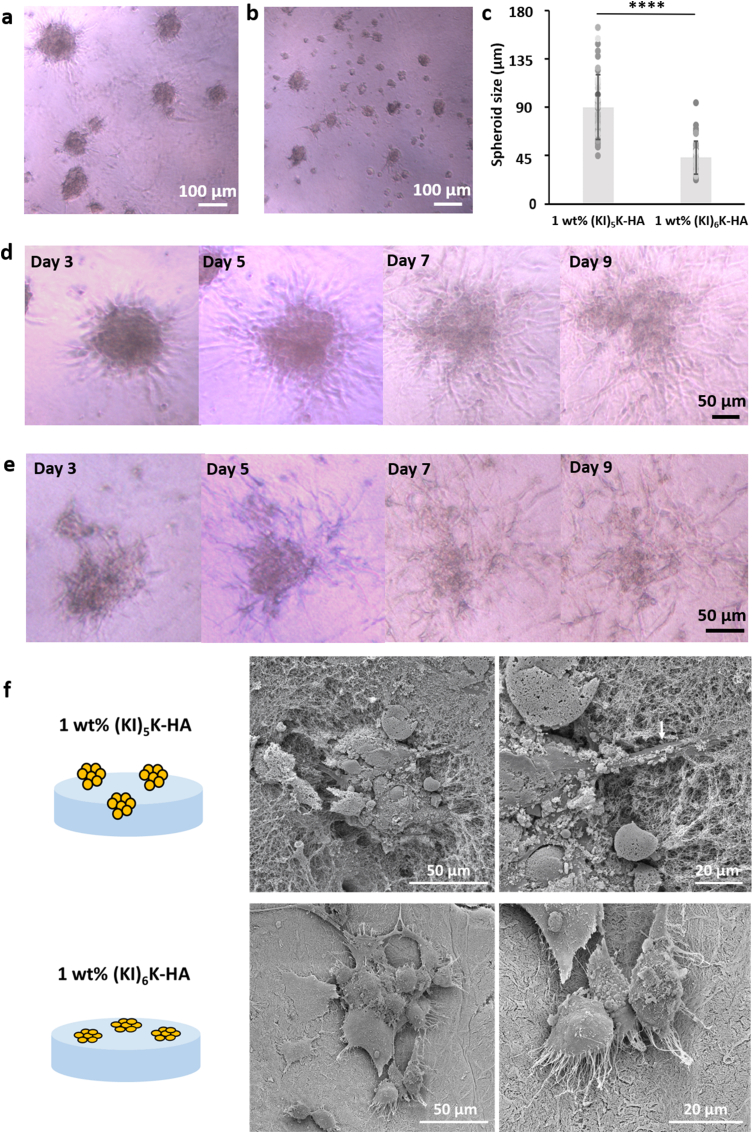
MSC spheroids formed on (a) 1 ​wt% (KI)_5_K-HA and (b) 1 ​wt% (KI)_6_K-HA hydrogels observed by optical microscopy on day 3, and (c) analysis of spheroid size (∗∗∗∗ ​= ​p ​< ​0.0001, and error bars represent standard deviation (n ​= ​40)). The disassembly of the MSC spheroids on (d) 1 ​wt% (KI)_5_K-HA and (e) 1 ​wt% (KI)_6_K-HA hydrogels was also observed over 9 days. (f) SEM images of MSC assemblies on supramolecular peptide-HA hydrogels on day 7.

**Fig. 6:**
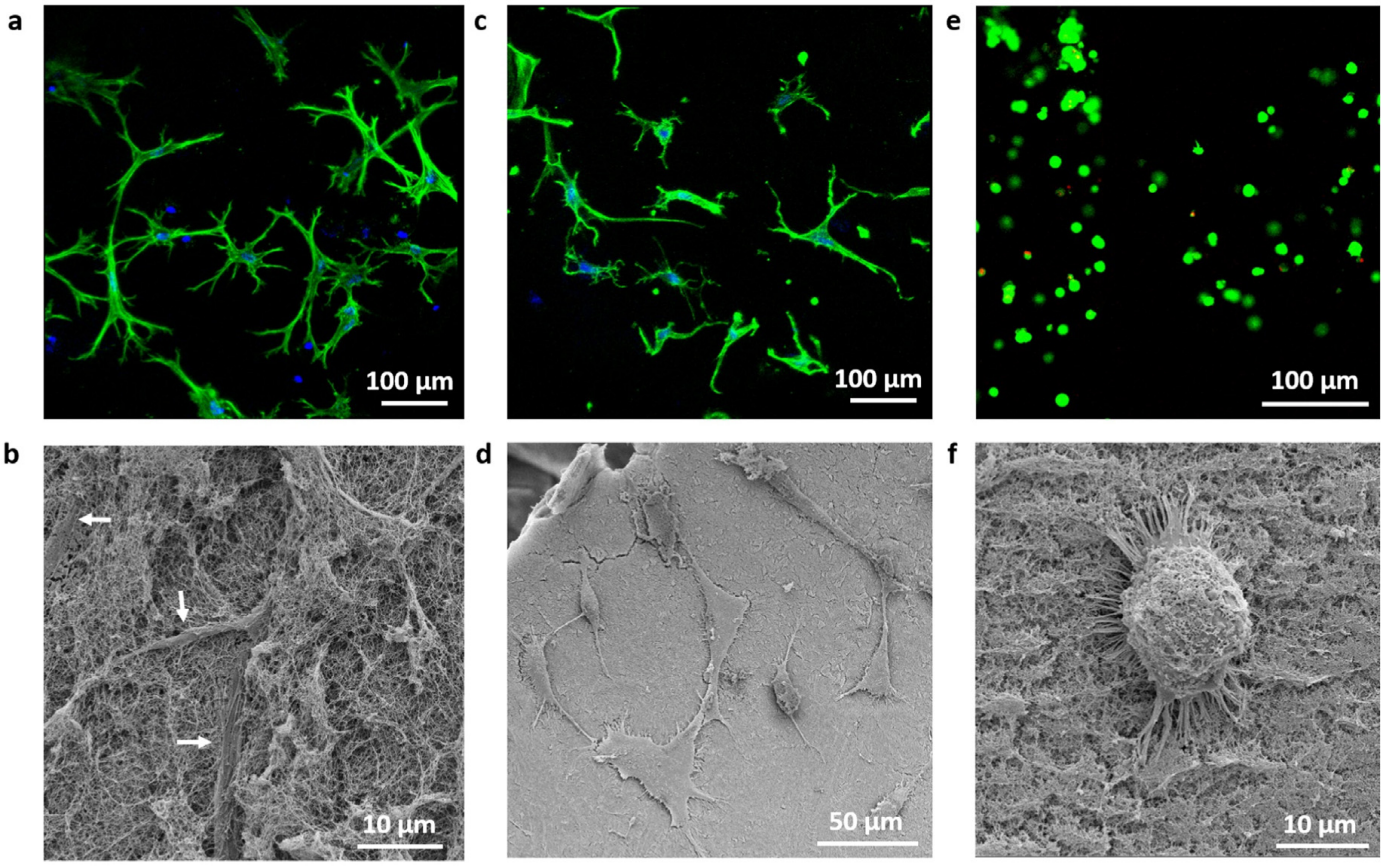
CLSM and SEM images of disassociated MSCs cultured on (a, b) (KI)_5_K-HA and (c, d) (KI)_6_K-HA hydrogels. F-actin was labelled with FITC-phalloidin (green) and nuclei with DAPI (blue). (e) CLSM image of calcein-stained live cells (green) and ethidium homodimer-stained dead cells (red) and (f) SEM image of round cell on (KI)_5_K-alginate hydrogels on day 7. (For interpretation of the references to colour in this figure legend, the reader is referred to the Web version of this article.)

Having confirmed the stability of the supramolecular (KI)_4_K-HA, (KI)_5_K-HA and (KI)_6_K-HA hybrid hydrogels in cell culture media (DMEM), cell culture on the hydrogels was undertaken. MSCs, trypsinized from the standard 2D adherent cell culture, were first seeded on top of 1 ​wt% (KI)_4_K-HA, 1 ​wt% (KI)_5_K-HA and 1 ​wt% (KI)_6_K-HA hydrogels. However, the cell-seeded 1 ​wt% (KI)_4_K-HA hydrogels were not stable and easily collapsed. On both 1 ​wt% (KI)_5_K-HA and 1 ​wt% (KI)_6_K-HA hydrogel (Fig. 5a and b), cell spheroids were observed on day 3. The diameter of the spheroids on 1 ​wt% (KI)_5_K-HA hydrogel is 89.0 ​± ​30.1 ​μm, which appears to be significantly larger than in the 1 ​wt% (KI)_6_K-HA hydrogel (42.2 ​± ​15.2 ​μm) (Fig. 5c). In some of the formed spheroids, cells show different morphology in the exterior and interior, with cells on the border of the spheroids being more elongated and extended. Additionally, some elongated cells on the border would gradually migrate out of the spheroid in the following days (Fig. 5d and e), which is commonly observed in MSC spheroids when maintained in the growth media [52,53]. Reduced diffusion of nutrients, oxygen, and waste is expected in the cell spheroids, with aggregated cells creating spatial hindrance, and thus compromising the viability of cells, especially the cells at the core of the spheroids [29]. The viability of MSCs in the spheroids was examined and good viability was observed for dissociated cells and cells in the spheroids on both peptide-HA hydrogels with an evident morphology change of the cells over time (Fig. S12, Supplementary data). SEM images were obtained on day 7 to observe the interaction between the cell spheroid and the hydrogel substrate (Fig. 5f). The interaction of the MSC spheroids and the hydrogel surface is expected to play a role in the spheroid disassembly, as MSC spheroids cultured in non-adhesive hydrogel would not dissociate and would remain spherical with the smooth edges, while cells in adhered spheroids may migrate out [54,55]. In the 1 ​wt% (KI)_5_K-HA hydrogel, MSC spheroids were found trapped inside the hydrogel, where some of the cells were extending outward and entangling with nanofibers of the hydrogel at the edge of the spheroid (white arrow), while round cells could also be observed inside the spheroid. It is known that cells at the core of the cell spheroid often display spherical morphology, with the size significantly smaller than in 2D monolayer culture [29]. While for 1 ​wt% (KI)_6_K-HA hydrogel MSC assemblies were found on the surface of the hydrogel, the formation of filopodia at the outskirt of the cell assembly was also observed. Filopodia on the edge of cells is typically prominent during initial cell adhesion on 2D substrate and might also indicate the possibility of further cell migration [56,57]. The existence of filopodia may suggest cells were migrating out of the cell assemblies and adhering to the hydrogel surface on day 7. Interestingly, cell assemblies on 1 ​wt% (KI)_6_K-HA hydrogel display the typical cell behavior of both 2D (presence of filopodia and relatively higher flattening degree) and 3D (cells aggregated and stacked), which is likely caused by the ‘competition’ between cell-substrate and cell-cell adhesion, as the cell aggregation fate is determined by both cell-substrate and cell-cell adhesion interactions [58]. Many disassociated cells were also observed on day 7, mainly cells migrating out of the spheroid and cells not integrating into the spheroids. They exhibit spindle-like or spreading morphology, indicating good cell adhesion. Disassociated cells on 1 ​wt% (KI)_5_K-HA hydrogel (Fig. 6a and b, white arrows) were not completely visible as they were entrapped in the hydrogel, while cells on the 1 ​wt% (KI)_6_K-HA hydrogel were clearly residing on the hydrogel surface (Fig. 6c and d). As MSC spheroids are favorably formed in non-adhesive material, it is assumed that (KI)_n_K-HA hydrogels may be non-cell-adhesive initially, but they may rearrange due to their dynamic supramolecular nature and favor spheroid disassembly as adhesion of disassociated cells could also be observed for later culture time. It is apparent that peptide-HA hydrogel could support both spheroid formation and cell disassociation, indicating the interaction between MSCs and peptide-HA hydrogel might change over the time. The mechanism of MSCs spheroid formation and disassembly on the supramolecular peptide-HA hydrogel still requires further investigation. Comparing to state-of-the art culture of spheroids in 3D (cells encapsulated within a gel material), spheroid culture on the surface of hydrogels facilitates the retrieval of the spheroid masses for characterization purposes and/or further use in regenerative medicine applications (e.g. cell therapy). MSCs were also cultured on 2 ​wt% (KI)_5_K-HA and 2 ​wt% (KI)_6_K-HA hydrogels. Despite cell aggregation tendency was observed, no larger spheroids were formed (Fig. S13, Supplementary data). Though 2 ​wt% (KI)_6_K-HA hydrogel in DMEM showed much higher stiffness than 1 ​wt% (KI)_6_K-HA hydrogel (Fig. S14, Supplementary data), 2 ​wt% (KI)_5_K-HA hydrogel in DMEM had similar rheological profile with 1 ​wt% (KI)_5_K-HA hydrogel. Since the 2 ​wt% (KI)_5_K-HA hydrogel did not support the formation of cell spheroids, it is likely that mechanical properties may not be the only factor affecting the ability of the hydrogel for promoting spheroid formation.

To gain further insights on the mechanism of cell spheroid formation on the peptide-HA hydrogels, supramolecular peptide-alginate hydrogels were also utilized for the seeding of MSCs and explore the contribution of the hydrogel composition to the spheroid formation on the peptide-HA system. Alginate is an anionic polysaccharide extracted from seaweeds and could self-assemble with (KI)_n_K peptides to form hybrid hydrogels similar to the peptide-HA system. MSCs seeded on supramolecular peptide-alginate hydrogel (1 ​wt% (KI)_4_K-alginate, 1 ​wt% (KI)_5_K-alginate and 1 ​wt% (KI)_6_K-alginate) remained round during 7 days of culture, but showed little aggregation tendency (Fig. 6e, Fig. S15, Supplementary data). Therefore, it is postulated that HA plays an essential role in the formation of the MSC spheroids on the supramolecular peptide-HA hydrogels. HA is known to have a superhydrophilic character due to the presence of carboxylate (-COO^-^) and hydroxyl (-OH) groups, which limits protein absorption, reduce cell adhesion and promote cell aggregation [59]. The formation of MSCs spheroids on HA-chitosan coated surfaces was reported by Huang et al. where CD44-HA interaction was believed to be important for the spheroid formation [53]. In order to probe the possible function of (KI)_n_K peptide in mediating MSC behavior, Ca^2+^-alginate hydrogel (alginate ionically crosslinked by Ca^2+^) was used for direct comparison with peptide-alginate hydrogel. In fact, Ca^2+^-alginate hydrogel is well known to be inert to adhesive cells [54,60]. MSCs on peptide-alginate hydrogel exhibited better adhesion than on Ca^2+^-alginate hydrogel (Fig. S16, Supplementary data). SEM images at higher magnification showed filopodia and lamellipodia at the edge of the round cell (Fig. 6f), indicating cell-substrate interaction on the peptide-alginate hydrogel. It is hypothesized that electrostatic interactions between the cationic peptides and anionic serum proteins and the cell surface are likely to facilitate the attachment of cells. However, the round morphology of MSCs suggested that cell adherence on peptide-alginate hydrogel surface is less prominent, compared to the dissociated cells on peptide-HA hydrogels and on conventional 2D cell culture, where MSCs exhibit typical elongated morphology. Taken together, these results indicate that peptide-HA hydrogels offer a set of properties suitable for modulating the cell microenvironment.

## 4 Conclusions

Novel supramolecular hyaluronan (HA) hydrogels were easily fabricated via self-assembly of cationic β-sheet peptides [(KI)_n_K] without requiring HA chemical modification and the use of additional chemical cross-linkers. The hybrid hydrogels exhibited viscoelastic behavior with adjustable stiffness and varied morphologies, from homogeneous to microporous and hierarchical structures. The diverse structural and mechanical properties of the hydrogels were regulated by the peptide length (charge/hydrophobic ratio and β-sheet forming ability) and concentration, providing new ways and exquisite insights to create biomimetically structured hydrogels. Culture of MSCs on the peptide-HA hydrogels led to spontaneous cell spheroid formation, expanding the MSC spheroid culture platforms while offering the possibility to direct cell-cell and cell-microenvironment interactions for enhanced therapies in regenerative medicine applications. Likewise, the use of the structured peptide-HA hydrogels to grow tumorspheres and create *in vitro* tumor models is also anticipated and highly relevant, as HA is known to be abundant in the tumor microenvironment of several cancers and regulate the behavior of cancer cells.

## Credit author statement

**YY**: performing the majority of the experimental work; analysis of data; writing the original draft; reviewing and editing the various versions of the manuscript. **YS**: contribution to the peptide characterization work; reading and reviewing the various versions of the manuscript. **JB**: conceptualization; reading and reviewing the various versions of the manuscript. **AM**: formal analysis and interpretation of SAXS data; reading and reviewing the various versions of the manuscript. **HSA**: conceptualization; funding acquisition, supervision of **YY**, **YS** and **JB**'s work, reviewing and editing the various versions of the manuscript.

## Declaration of competing interest

The authors declare that they have no known competing financial interests or personal relationships that could have appeared to influence the work reported in this paper.

## Data Availability

Data will be made available on request.
