# Supplementary material for: Structuring supramolecular hyaluronan hydrogels via peptide self-assembly for modulating the cell microenvironment
Source: Mater Today Bio. 2023 Mar 2;19:100598. doi: 10.1016/j.mtbio.2023.100598 (PMC10024175; doi:10.1016/j.mtbio.2023.100598)
Supplement: Multimedia component 1 [file mmc1.docx]

**Supplementary data**

**Structuring supramolecular hyaluronan hydrogels via peptide self-assembly for modulating the cell microenvironment**

*Yichen Yuan ^a,b^, Yejiao Shi ^a,c^, Jayati Banerjee ^a^, Amin Sadeghpour ^d^, and Helena S. Azevedo ^a,e,f,^**

^a^ School of Engineering and Materials Science & Institute of Bioengineering, Queen Mary

University of London, London, E1 4NS, UK

^b^ Zhejiang Lab, Hangzhou 311121, Zhejiang, Peoples R China

^c^ Institute of Translational Medicine, Shanghai University, Shanghai 200444, Peoples R China

^d^ School of Food Science and Nutrition, University of Leeds, Leeds, LS2 9JT, UK

^e^ i3S - Instituto de Investigação e Inovação em Saúde, Universidade do Porto, Portugal

^f^ INEB - Instituto de Engenharia Biomédica, Universidade do Porto, Rua Alfredo Allen, 208, 4200-180 Porto, Portugal

Email: [h.azevedo@qmul.ac.uk](mailto:h.azevedo@qmul.ac.uk); [hazevedo@i3s.up.pt](mailto:hazevedo@i3s.up.pt)

**Table S1** Molecular information and purity of the peptides synthesized and purified in this work.

| Peptide | Chemical formula | Expected mass (g/mol) | Observed mass  (g/mol) | Purity^a^  (%) | Retention time (min) |
| --- | --- | --- | --- | --- | --- |
| (KI)_2_K | C_32_H_63_N_9_O_6_ | 669.49 | 670.86 [M+H]^+^  336.20 [M+2H]^2+^  224.41 [M+3H]^3+^ | 95 | 7.5 |
| (KI)_3_K | C_44_H_86_N_12_O_8_ | 910.67 | 912.21 [M+H]^+^  456.83 [M+2H]^2+^  304.98 [M+3H]^3+^ | 94 | 8.5 |
| (KI)_4_K | C_56_H_109_N_15_O_10_ | 1152.58 | 577.49 [M+2H]^2+^  385.45 [M+3H]^3+^  289.26 [M+4H]^4+^ | 99 | 9.0 |
| (KI)_5_K | C_68_H_132_N_18_O_12_ | 1393.92 | 349.68 [M+3H]^3+^  465.91 [M+4H]^4+^ | 97 | 9.5 |
| (KI)_6_K | C_80_H_155_N_21_O_14_ | 1634.21 | 546.32 [M+3H]^3+^  410.11 [M+4H]^4+^  328.20 [M+5H]^5+^ | 98 | 9.9 |

^a^ Determined by HPLC


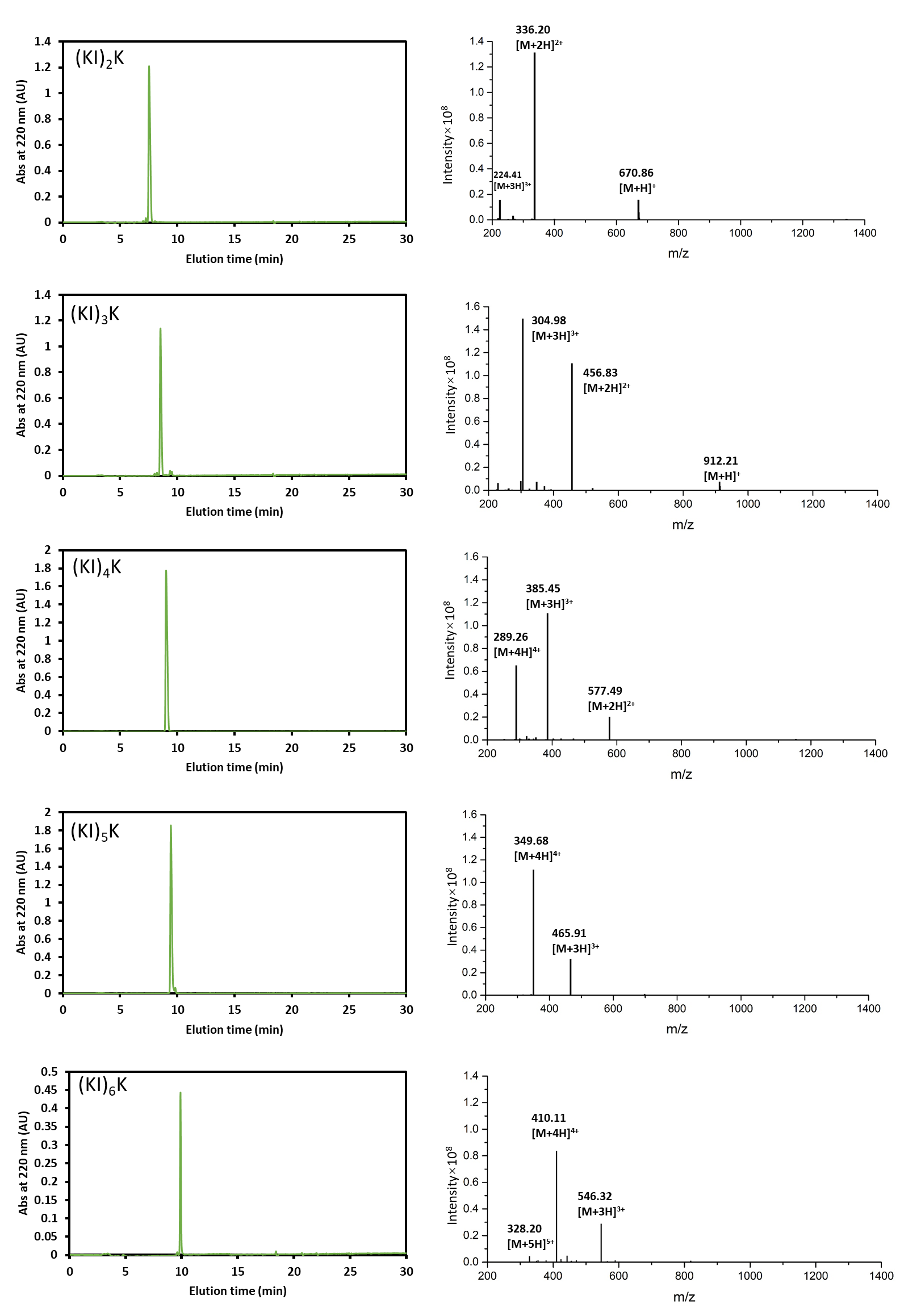


**Figure S1:** Analytical RP-HPLC traces and ESI mass spectra of purified (KI)_n_K peptides. Peptides are eluted in a gradient of water/acetonitrile (0.1 % TFA).


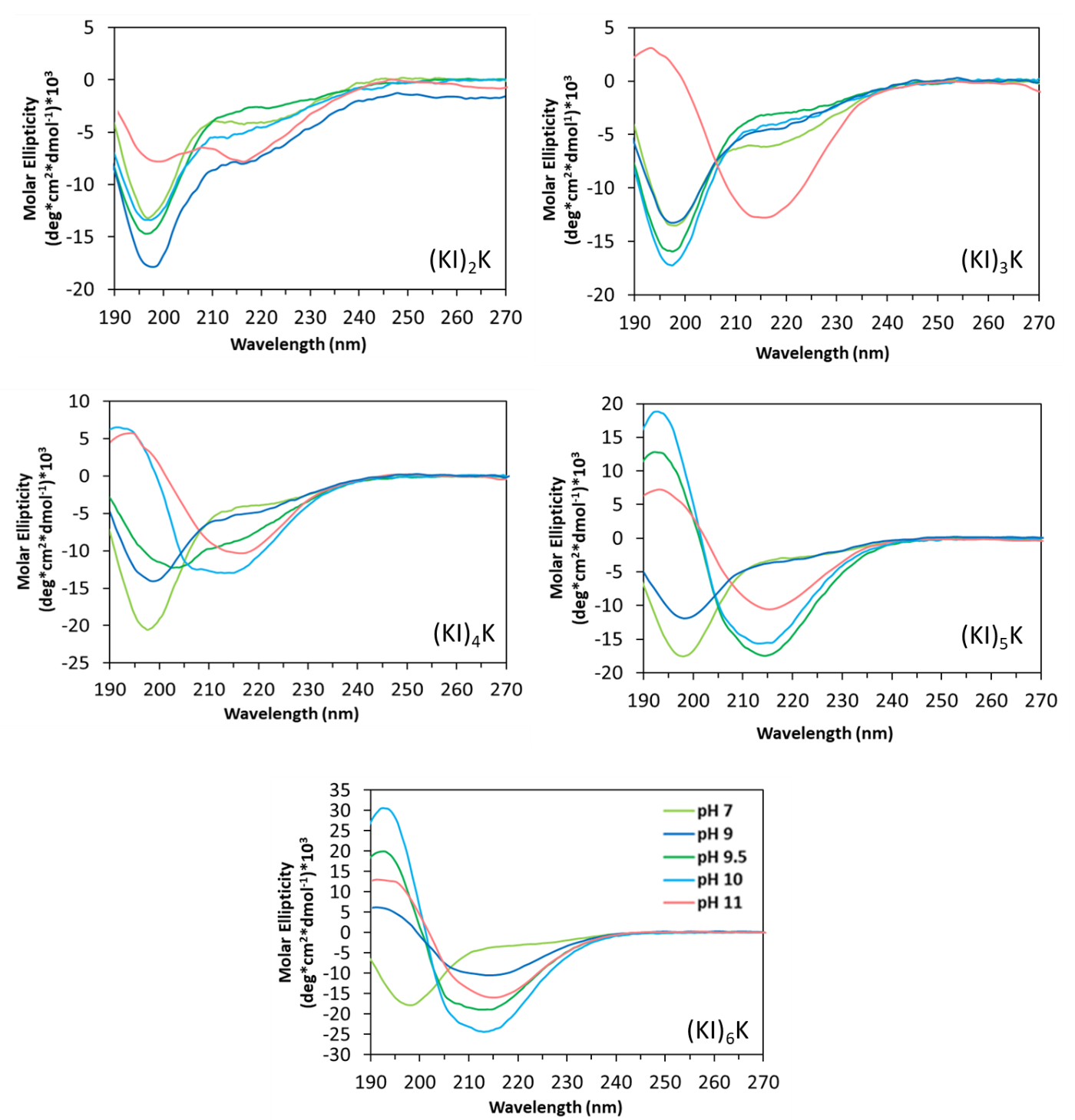


**Figure S2:** CD spectra of (KI)_n_K peptide solutions (0.1 mM) at pH 7, 9, 9.5 10 and 11.


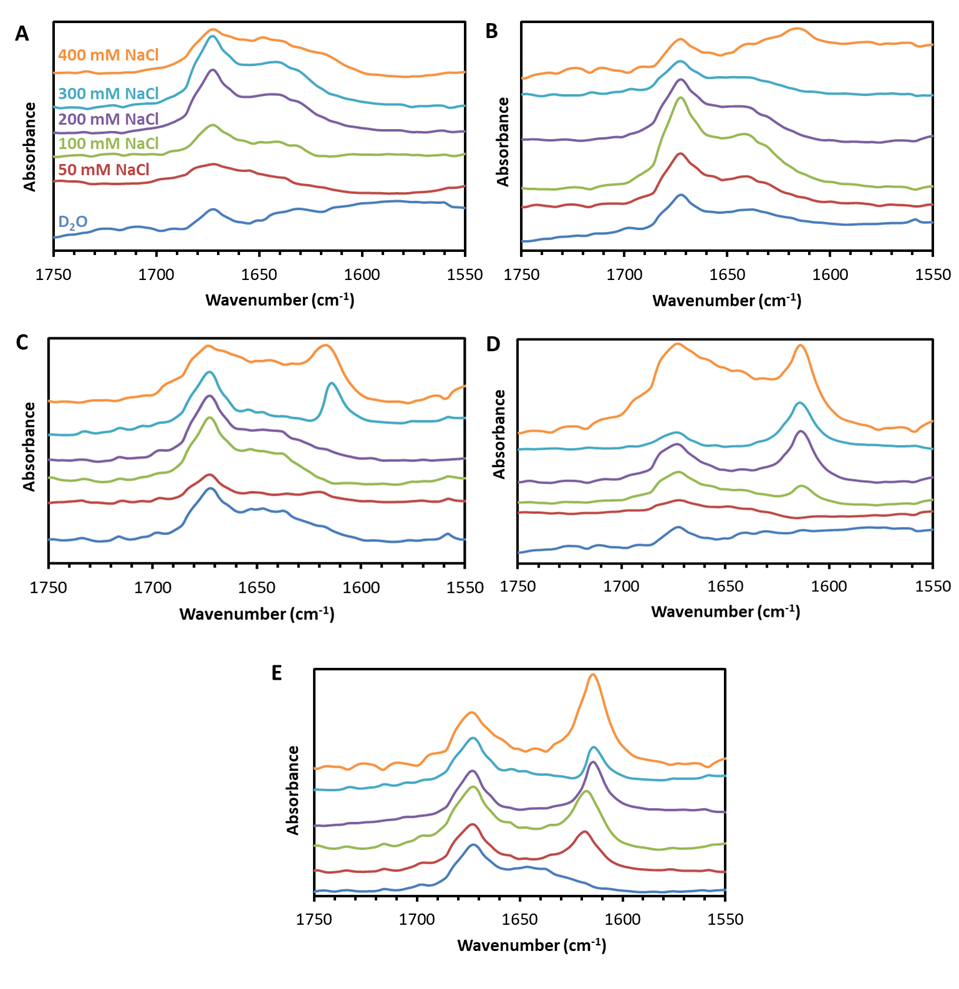


**Figure S3.** FTIR spectra of (A) (KI)_2_K, (B) (KI)_3_K, (C) (KI)_4_K, (D) (KI)_5_K and (E) (KI)_6_K peptide solutions with different NaCl concentration at pH 7.


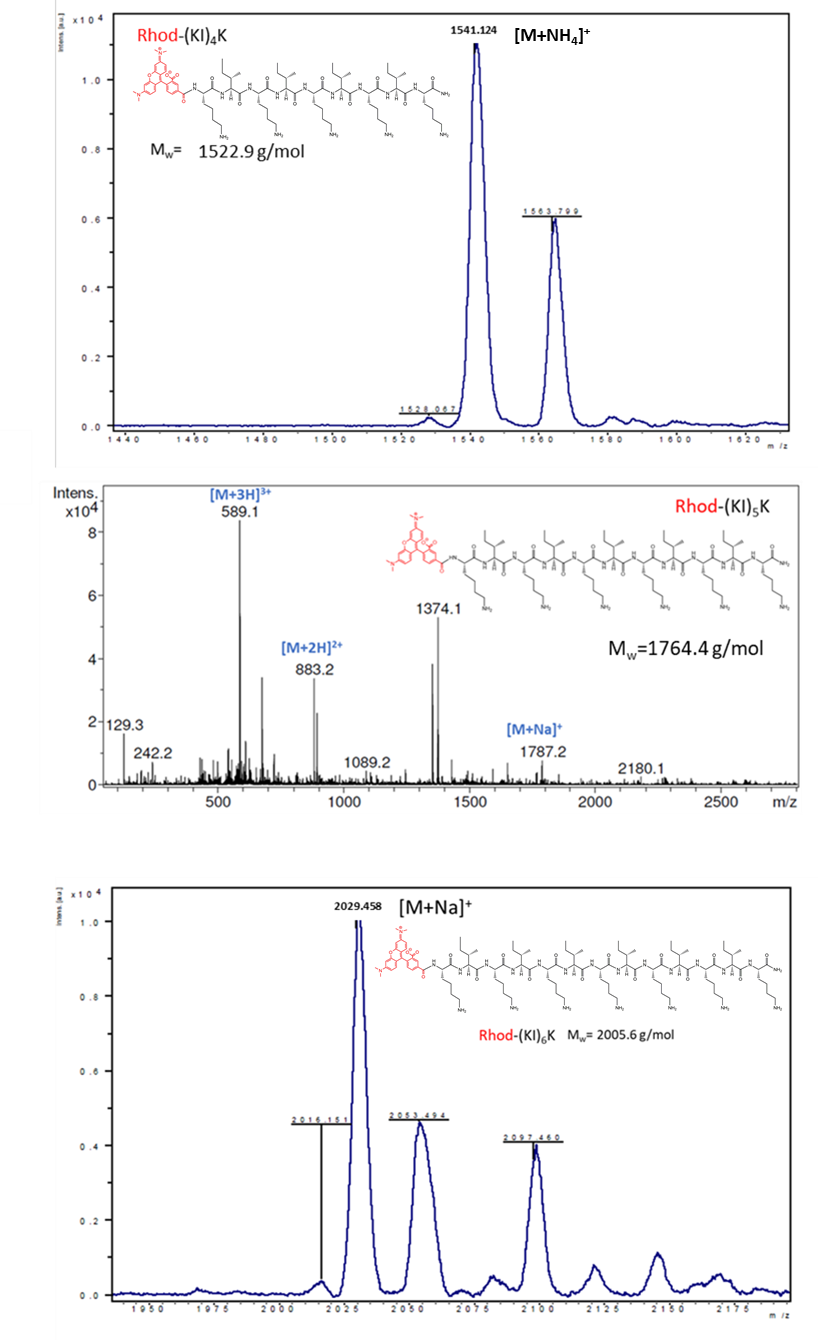


**Figure S4.** The mass spectra and chemical structures of Rhod-(KI)_4_K (obtained by MALDI-TOF MS), Rhod-(KI)_5_K (obtained by ESI MS) and Rhod-(KI)_6_K (obtained by MALDI-TOF MS).


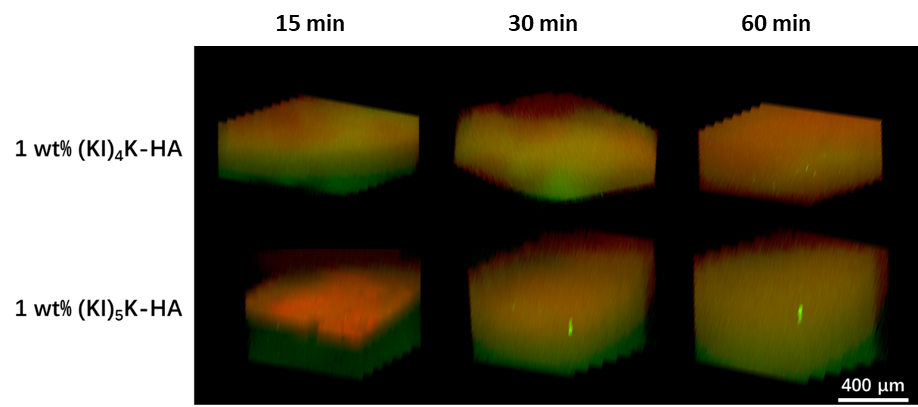


**Figure S5.** Reconstructed 3D images of CLSM used to study the kinetic of single-phase 1 wt% (KI)_4_K-HA and 1 wt% (KI)_5_K-HA complex.

(C)
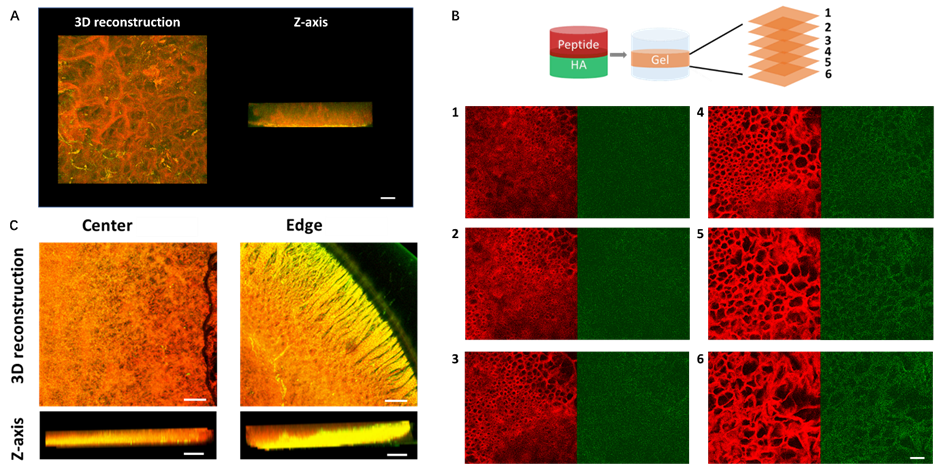


**Figure S6.** CLSM images of 2 wt% (KI)_5_K-HA (A, B) and 2 wt% (KI)_6_K-HA hydrogels after overnight incubation in salt-free condition. Scale bars for images in A and B are 200 µm and for C are 100 µm.


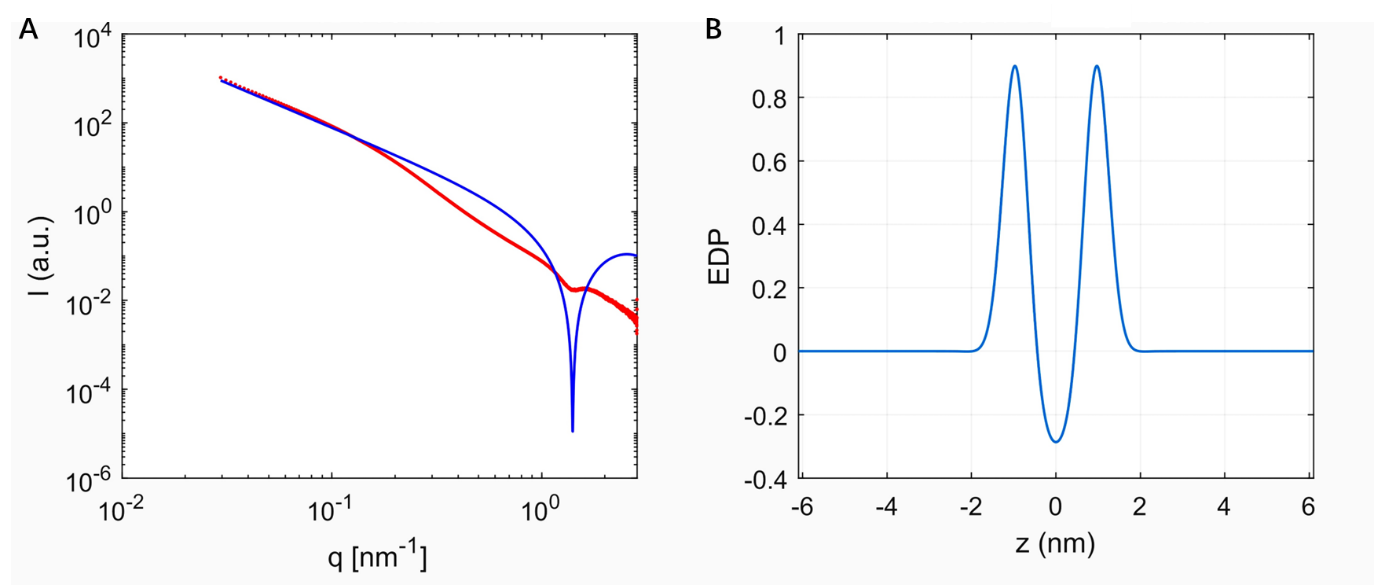


**Figure S7**. (A) The simulated scattering curve (blue) based on lamellar self-assembly with the bilayer deviating from 2 wt% (KI)_5_K-HA hydrogel scattering curve (red). (B) The electron density profile associated to the simulated curve.


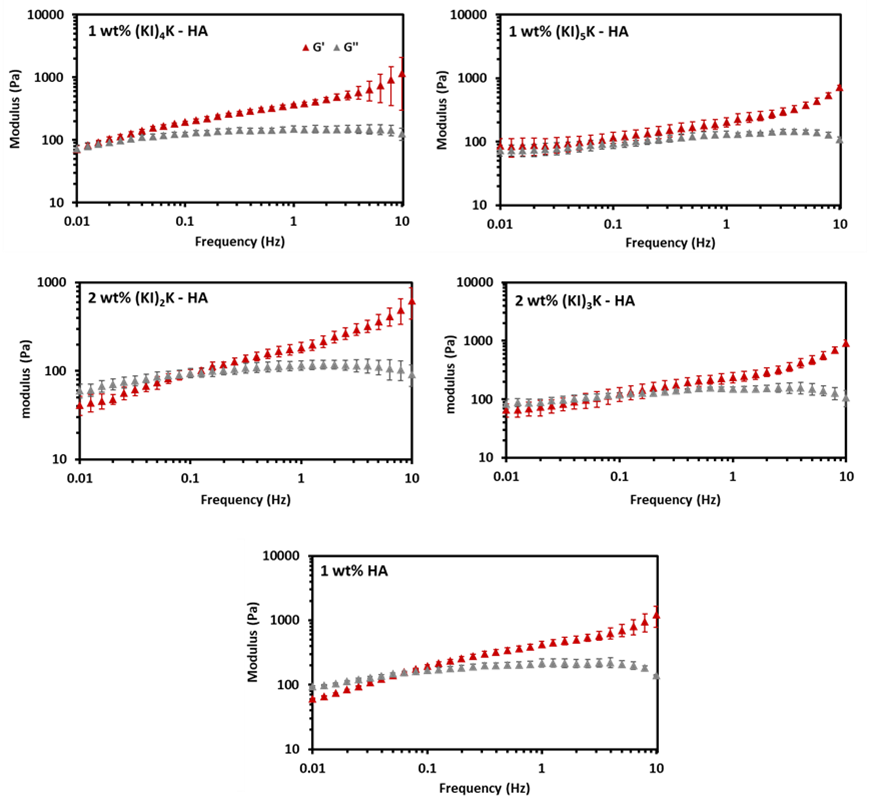


**Figure S8.** Rheological analysis of single-phase (KI)_n_K-HA complexes and 1 wt% HA solution. Standard deviation is indicated by error bars and measurements were performed in triplicate.


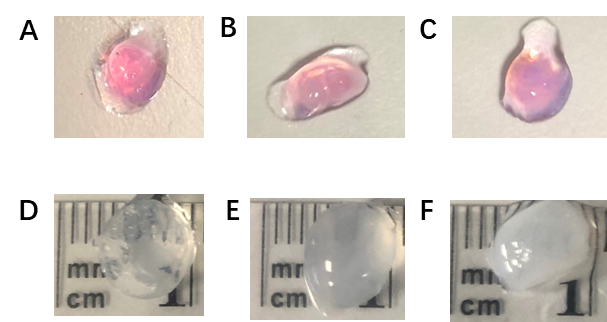


**Figure S9.** Self-supporting hydrogels formed by (A) 1 wt% (KI)_4_K-HA (B) 1 wt% (KI)_5_K-HA and (C) 1 wt% (KI)_6_K-HA with the addition of cell culture media (DMEM) and washed in PBS. (D) 2 wt% (KI)_4_K-HA (E) 2 wt% (KI)_5_K-HA and (F) 2 wt% (KI)_6_K-HA hydrogels formed in PBS.


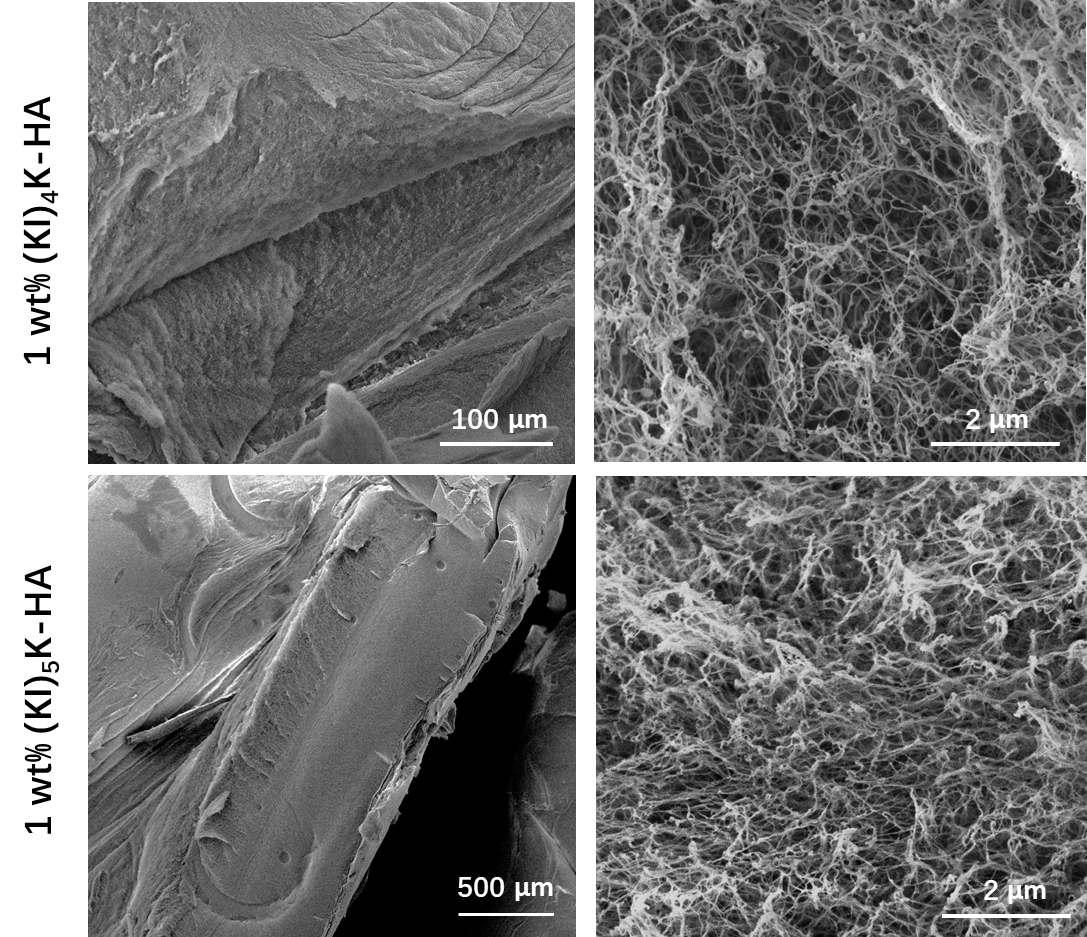


**Figure S10.** SEM images show homogeneous cross-section of single-phase 1 wt% (KI)_4_K-HA and 1 wt% (KI)_5_K-HA nanofibrous hydrogels incubated in PBS.


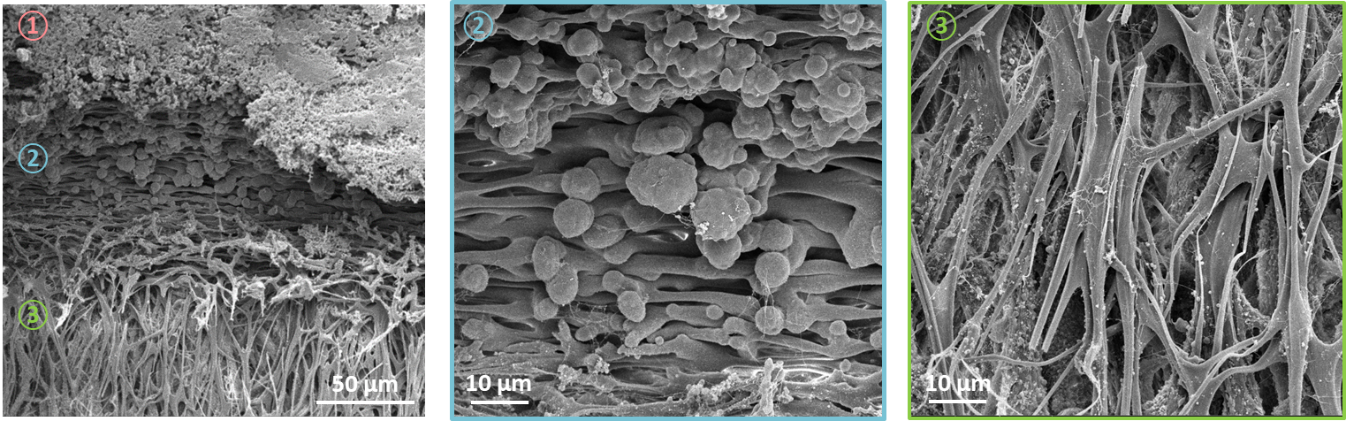


**Figure S11** SEM images showing heterogeneous structures at the edge of 1 wt% (KI)_6_K-HA hydrogel composed of three different zones.


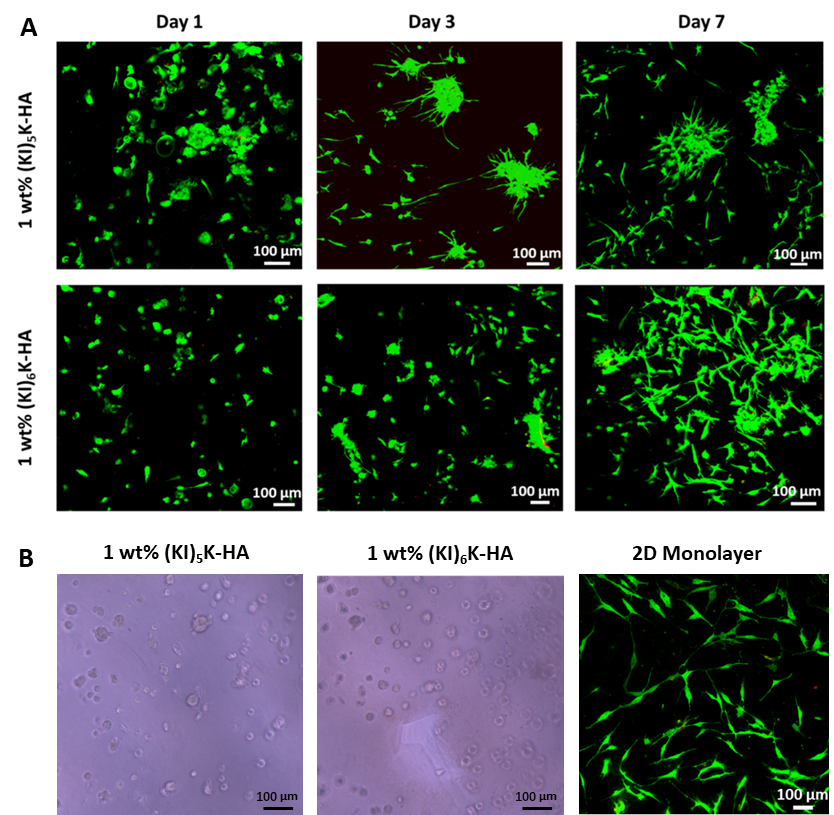


**Figure S12.** (A) CLSM of calcein-stained live MSCs (green) and ethidium homodimer-stained dead cells (red) on peptide-HA hydrogels. Dissociated cells and cell spheroids on both peptide-HA hydrogels show good viability and a morphology change of the cells is also evident over time. (B) Optical microscopy (left and center) and CLSM (live/dead assay, right) images taken on day 1showing single cells adopting a round shape when seeded on the peptide-HA hydrogels, but also small cell spheroids, in contrast to the standard 2D monolayer culture, where MSCs often exhibit spindle-like morphology.


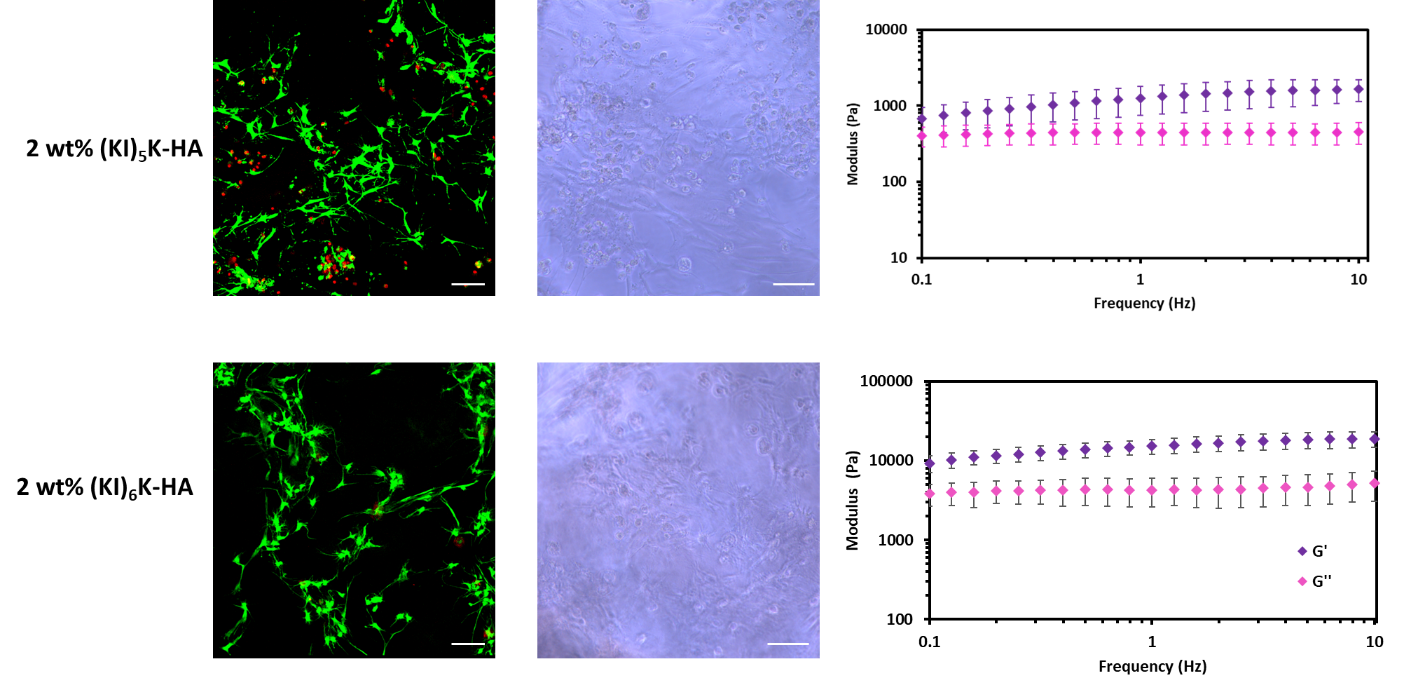


**Figure S13.** CLSM (live/dead assay) and optical microscopy images (left and center, scale bar = 100 μm) of MSCs cultured on 2 wt% (KI)_5_K-HA and 2 wt% (KI)_6_K-HA hydrogels on day 3, and rheology of both hydrogels after incubation in DMEM. Error bars represent standard deviation (n = 3).

***
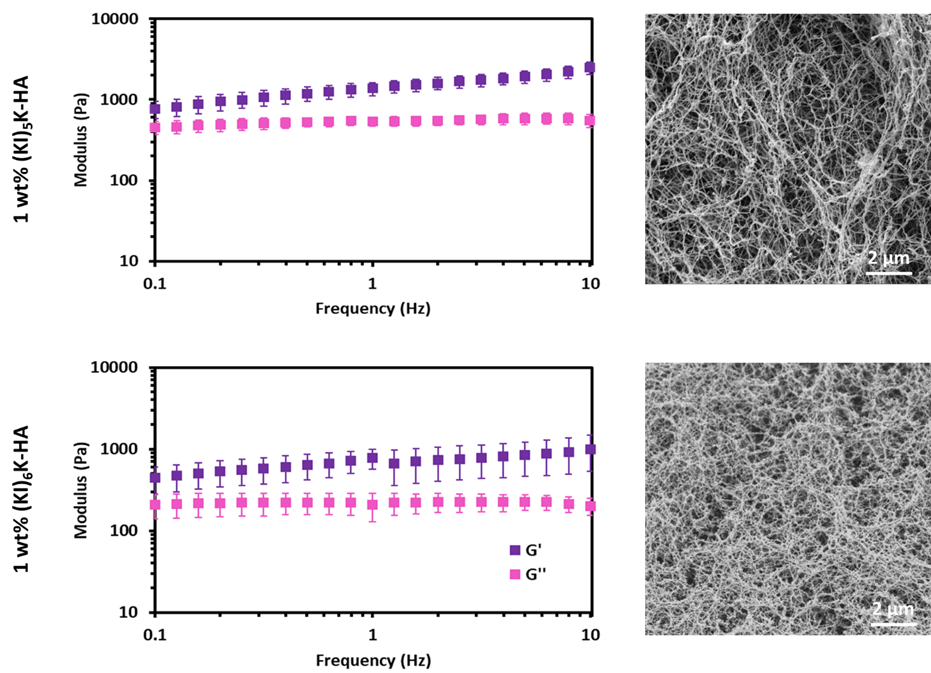
***

**Figure S14.** Bulk rheology and SEM images of the surfaces of 1 wt% (KI)_5_K-HA and 1 wt% (KI)_6_K-HA cell-free hydrogels. Both hydrogels were incubated in DMEM with 10% FBS overnight. Error bars represent standard deviation (n = 3). 1 wt% (KI)_5_K-HA hydrogel has a surface with looser structure (less dense nanofibers) than 1 wt% (KI)_6_K-HA hydrogel, which probably leads to the cell spheroid entrapped in the hydrogel.


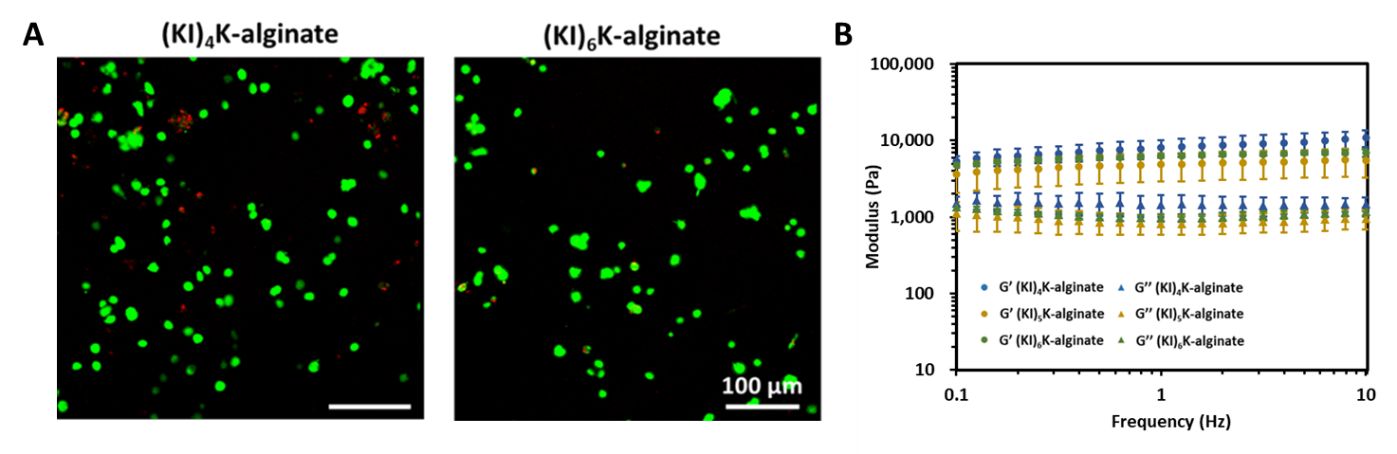


**Figure S15.** (A) CLSM of calcein-stained live cells (green) and ethidium homodimer-stained dead cells (red) on (KI)_4_K-alginate and (KI)_6_K-alginate hydrogels on day 7. (B) Rheological analysis of (KI)_n_K-alginate hydrogels incubated in DMEM with 10% FBS overnight. Error bars represent standard deviation (n = 3).


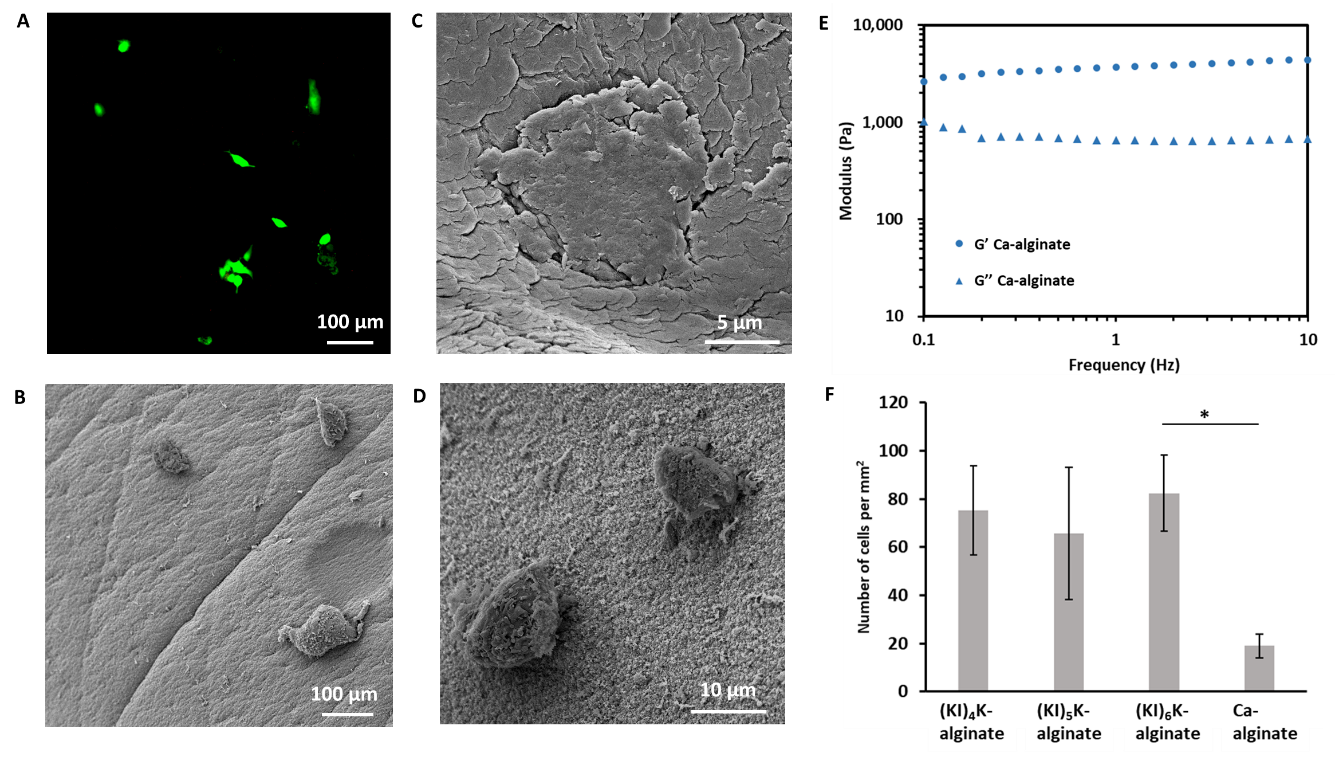


**Figure S16**. (A) CLSM of calcein-stained live cells (green) and ethidium homodimer-stained dead cells (red) on Ca^2+^-alginate hydrogels on day 7. (B-D) SEM images of MSCs on Ca-alginate hydrogels on day 7. (E) Bulk rheology measurement of 2 wt% Ca^2+^-alginate cell-free hydrogels incubated in DMEM with 10% FBS overnight. (F) The analysis of cell density on peptide-alginate and Ca^2+^-alginate hydrogels. (*=p < 0.0332, and error bars represent standard deviation (n = 3)).
